# RGCC-mediated PLK1 activity drives breast cancer lung metastasis by phosphorylating AMPKα2 to activate oxidative phosphorylation and fatty acid oxidation

**DOI:** 10.1186/s13046-023-02928-2

**Published:** 2023-12-15

**Authors:** Shaojie Cheng, Xueying Wan, Liping Yang, Yilu Qin, Shanchun Chen, Yongcan Liu, Yan Sun, Yuxiang Qiu, Luyi Huang, Qizhong Qin, Xiaojiang Cui, Mingjun Wu, Manran Liu

**Affiliations:** 1 Key Laboratory of Laboratory Medical Diagnostics, Chinese Ministry of Education, Chongqing Medical University, No.1, Yi-Xue-Yuan Road, Yu-Zhong District, Chongqing, 400016 China; 2 Department of Laboratory Medicine, the Second Affiliated Hospital of Chongqing Medical University, Chongqing, 400010 China; 3 Chongqing Key Laboratory of Sichuan-Chongqing Co-Construction for Diagnosis and Treatment of Infectious Diseases Integrated Traditional Chinese and Western Medicine, Chongqing Hospital of Traditional Chinese Medicine, Chongqing, 400021 China; 4 Department of Cell Biology and Medical Genetics, Basic Medical School, Chongqing Medical University, Chongqing, 400016 China; 5 The Key Laboratory of Molecular Biology of Infectious Diseases Designated By the Chinese Ministry of Education, Chongqing Medical University, Chongqing, 400016 China; 6 Experimental Teaching Center of Basic Medicine Science, Chongqing Medical University, Chongqing, 400016 China; 7 Department of Surgery, Department of Obstetrics and Gynecology, Samuel Oschin Comprehensive Cancer Institute, Cedars-Sinai Medical Center, Los Angeles, CA 91006 USA; 8 Institute of Life Science, Chongqing Medical University, No.1, Yi-Xue-Yuan Road, Yu-Zhong District, Chongqing, 400016 China

**Keywords:** RGCC, Lung metastasis, PLK1, OXPHOS, Fatty acid oxidation

## Abstract

**Background:**

More than 90% of the mortality of triple-negative breast cancer (TNBC) patients is attributed to cancer metastasis with organotropism. The lung is a frequent site of TNBC metastasis. However, the precise molecular mechanism for lung-specific metastasis of TNBC is not well understood.

**Methods:**

RNA sequencing was performed to identify patterns of gene expression associated with lung metastatic behavior using 4T1-LM3, MBA-MB-231-LM3, and their parental cells (4T1-P, MBA-MB-231-P). Expressions of RGCC, called regulator of cell cycle or response gene to complement 32 protein, were detected in TNBC cells and tissues by qRT-PCR, western blotting, and immunohistochemistry. Kinase activity assay was performed to evaluate PLK1 kinase activity. The amount of phosphorylated AMP-activated protein kinase α2 (AMPKα2) was detected by immunoblotting. RGCC-mediated metabolism was determined by UHPLC system. Oxidative phosphorylation was evaluated by JC-1 staining and oxygen consumption rate (OCR) assay. Fatty acid oxidation assay was conducted to measure the status of RGCC-mediated fatty acid oxidation. NADPH and ROS levels were detected by well-established assays. The chemical sensitivity of cells was evaluated by CCK8 assay.

**Results:**

RGCC is aberrantly upregulated in pulmonary metastatic cells. High level of RGCC is significantly related with lung metastasis in comparison with other organ metastases. RGCC can effectively promote kinase activity of PLK1, and the activated PLK1 phosphorylates AMPKα2 to facilitate TNBC lung metastasis. Mechanistically, the RGCC/PLK1/AMPKα2 signal axis increases oxidative phosphorylation of mitochondria to generate more energy, and promotes fatty acid oxidation to produce abundant NADPH. These metabolic changes contribute to sustaining redox homeostasis and preventing excessive accumulation of potentially detrimental ROS in metastatic tumor cells, thereby supporting TNBC cell survival and colonization during metastases. Importantly, targeting RGCC in combination with paclitaxel/carboplatin effectively suppresses pulmonary TNBC lung metastasis in a mouse model.

**Conclusions:**

RGCC overexpression is significantly associated with lung-specific metastasis of TNBC. RGCC activates AMPKα2 and downstream signaling through RGCC-driven PLK1 activity to facilitate TNBC lung metastasis. The study provides implications for RGCC-driven OXPHOS and fatty acid oxidation as important therapeutic targets for TNBC treatment.

**Supplementary Information:**

The online version contains supplementary material available at 10.1186/s13046-023-02928-2.

## Introduction

Breast cancer is the most frequent malignancy in women worldwide, and triple-negative breast cancer (TNBC) is the most aggressive and associated with the worst prognosis among all breast cancer subtypes [1, 2]. More than 90% of the mortality of TNBC patients is attributed to cancer metastasis to different organ sites [3]. Lung is the most frequent site of TNBC metastasis [4, 5]. However, the precise mechanism of TNBC metastasis, particularly the critical signaling pathways driving the metastatic process, is not well understood [6]. Therefore, the identification and in-depth research of critical regulators of TNBC metastasis will be helpful for developing effective metastasis-targeting strategy and drug development.

Distant metastasis of tumor cells is an extremely challenging process, and only a small number of disseminated tumor cells (DTCs) can survive in the secondary site [6]. As an essential characteristic of malignancies, metabolic reprogramming plays a key role in cancer progression and metastasis [7]. These DTCs can evolve favorable traits essential for metastatic formation during dissemination and colonization through metabolic reprogramming [8]. Mitochondria, as the central player in cell energy metabolism, exert many crucial functions in tumor progression [9]. For instance, it has been reported that PGC-1α-mediated mitochondrial biogenesis and oxidative phosphorylation are prominent in breast cancer metastasis [10]. Distinct from primary prostate cancer cells, metastatic prostate cancer cells gradually turn to mitochondrial oxidative phosphorylation-dependent metabolism during pathological changes [11]. However, whether a common active mitochondrial metabolism exists in metastatic tumor cells, especially in organotropic metastasis, remains to be determined.

Response gene to complement 32 protein (RGCC), also known as RGC32, is a complement response gene [12]. The expression of RGCC can be stimulated and induced by numerous factors including complements, growth factors, cytokines, pro-angiogenesis factors, and hormones. It plays a major role in cell migration, differentiation, and fibrosis [13]. Additionally, RGCC promotes the entry of aortic smooth muscle cells into S-phase by directly binding to CDK1 and enhancing its kinase activity [14]. A previous study indicated that RGCC can improve glucose excursion under carbohydrate stress to maintain blood glucose homeostasis by regulating glucose metabolism-related genes, such as GFPT1, GLUT12, and GLP2R [15, 16]. The anomalous expression of RGCC has been observed in a variety of primary tumors, either enhancing or preventing the growth of tumors, due in part to the signaling context in tumor cells and the microenvironment [17]. In human lung cancer LTE cells, the downregulation of RGCC significantly reduced the proliferation, migration, and invasion of LTE cells [18]. Conversely, RGCC induced G2/M arrest and restrained the growth of glioma cells [19]. Of note, the functional role of RGCC in tumor metastasis has been scarcely reported.

In this study, we firstly revealed that RGCC was specifically upregulated in lung metastatic TNBC cells and played a key role in TNBC lung metastasis. RGCC protein in TNBC cells directly interacts with PLK1 and stimulates its kinase activity, which induces the phosphorylation of AMPKα2 to regulate mitochondrial oxidative phosphorylation (OXPHOS) and fatty acid oxidation, thereby promoting lung metastasis. We showed that targeting RGCC and its downstream signaling with the combined chemotherapy of paclitaxel and carboplatin suppressed lung metastasis, suggesting an effective therapeutic approach for treatment of metastatic TNBC.

## Materials and methods

### Clinical samples

The primary tumor tissue and lung metastasis tissue samples were obtained from TNBC patients with informed written consent in the First Affiliated Hospital of Chongqing Medical University, who had not undergone radiotherapy or chemotherapy. This study was approved by the Ethics Committee of Chongqing Medical University.

#### Cell culture

The mouse TNBC cell line 4T1 and the human TNBC cell lines MDA-MB-231, MDA-MB-468, BT-549, Hs578T and HCC1806 were obtained from the American Type Culture Collection (ATCC, USA). 4T1 and HCC1806 cells were cultured in RPMI 1640 medium (Gibco, USA) with 10% fetal bovine serum (FBS) (HyClone) and 1% penicillin/streptomycin (Beyotime, Shanghai, China). The other cells were cultured in DMEM medium (Gibco, USA) with 10% FBS and 1% penicillin/streptomycin. All cell lines were cultured in a 5% CO2 incubator at 37 °C.

#### Construction of tumor cell models

The lentivirus vectors expressing control shRNA and specific shRNA against human *RGCC* and murine *Rgcc* were synthesized by GenePharma (Shanghai, China). Constructs were transfected into HEK293T cells, and the resulting lentivirus supernatants were used to infect the third generation of lung-specific metastatic TNBC cells (4T1/LM3 and MDA-MB-231/LM3) with 4 g/ml polybrene, and stable cell lines were established by treating the infected cells with 800 μg/ml G418 (Beyotime, China) for two weeks. The target sequences against human *RGCC* and murine *Rgcc* and control shRNA used in this study are listed in Supplementary Table 1. For establishing RGCC overexpressing cells, *RGCC* expressing lentivirus were obtained from Genechem (Shanghai, China) and infected into TNBC cells (MDA-MB-468 and HCC1806). The infected cells were incubated with 1 μg/ml puromycin (Beyotime, China) to establish stable cells, following the manufacturer's instructions. To obtain the pGL3-*RGCC* wild-type (WT) or pGL3-*RGCC* mutant (MUT) reporter construct, the *RGCC* promoter containing the intact CEBPA binding sites and mutated binding sites were cloned into the pGL3 luciferase reporter vector (GenePharma, China). The pcDNA3.1 vector expressing CEBPA was obtained from GenePharma (Shanghai, China). Full-length of human PLK1 cDNA and its truncation variants were constructed and inserted into pcDNA3.1–3 × Flag vector (GeneCreate, Wuhan). The expression vectors including HA-RGCC, GST-RGCC, HA-PLK1 were constructed by GeneCreate (Wuhan, China). Genome editing of PLK1 was performed with the CRISPR/Cas9 system. Single guide RNA (sgRNA) oligonucleotides were cloned into U6-sgRNA-EF1a-Cas9-FLAG-P2A-puro packaged as lentiviruses (Genechem, Shanghai, China). The viruses were subjected to RGCC deficient and its control cells. The medium was changed to remove the virus 12 h after transfection, the transfection efficiency was tested after 72 h of culture, and then the dilution method was used to pick single clones. KO clones were identified by the Western blot. The PLK1 KO sgRNA sequence were: 5′- TTCCGGCGCGCCGAGTCCTT AGG- 3′. Mutations at T210A and T210D in the PLK construct were generated by PCR. The wild type and mutant PLK1s were amplified and subcloned into pLVX-CMV-blasticidin. Lentivirus expressing PLK1 was generated according to the manufacturer’s protocol. For viral infection, cells were treated with the viral particles and the infected cells were selected with 4 μg/ml blasticidin for one week.

#### Mouse experiments

Nude mice (female, 4–6 weeks old) were purchased from Hua Fukang Co. (Beijing, China) and used in animal experiments. All animal studies were approved by the Ethics Committee of Chongqing Medical University.

In vivo imaging was performed in mice injected with 4T1, MDA-MB-231, HCC1806 and MDA-MB-468 cells expressing luciferase (Hanbio, Shanghai, China). For the mouse models of organotropic metastasis, the luc-4T1 and luc-MDA-MB-231 cells were used as parental cells and were inoculated into the fourth mammary fat pads with 1 × 10^6^ cells/mouse. Then the mice with organ metastasis were selected for establishing primary culture of isolated cells from metastatic sites. When the cells formed stable clones, they were inoculated again into new mice to generate metastatic tumors. The highly organotropic metastasis cell lines were obtained after at least three rounds of such screening (named as 4T1/LM3 or MDA-MB-231/LM3 (lung-specific metastasis), 4T1/HM3 or MDA-MB-231/HM3 (liver-specific metastasis), 4T1/BM3 or MDA-MB-231/BM3 (brain-specific metastasis)).

For the experimental metastasis model, 1 × 10^6^ organotropic metastasis cells expressing luciferase in 100 μl PBS were injected into nude mice via the tail vein. Two months later, mice were sacrificed, and lung tissues were analyzed for the incidence of metastasis.

For bioluminescent imaging (BLI), mice were anesthetized and injected intraperitoneally with the substrate D-luciferin (150 mg/kg) and imaging pictures were taken by small animal living imaging system (Berthold LB983, Germany). The photon flux of each mouse was calculated by living image software version 2.50.

For drug treatment studies in the mice models, 1 × 10^6^ MDA-MB-231/LM3 cells in 100 µl PBS were inoculated into the fourth mammary fat pads of each mouse. Tumor sizes were measured every three days with digital calipers, when the primary tumor xenografts reached about 50 mm^3^, mice were intraperitoneally injected with 5 mg/kg of carboplatin (MCE; NSC 241240) and 10 mg/kg of paclitaxel (MCE; HY-B0015) every three days for three consecutive weeks. Then all experimental mice were euthanized, and mouse liver, spleen, and kidney tissues were taken out for weighing, and lung tissues were used for the analysis of metastasis.

#### RNA preparation, qRT-PCR, and RNA sequencing

Total RNA was isolated using the TRIzol reagent (Takara, Japan), and cDNA was generated using the PrimeScript RT Reagent Kit (Takara, Japan). The SYBR Premix Ex Taq II kit (Takara, Japan) was used for the quantitative real-time PCR. The primer sequences used in qRT-PCR are listed in Supplementary Table 2. RNA sequencing was carried out by Shengyin Biotech (Shanghai, China), and differential gene expression (DEG) analysis was performed using the DESeq2 package.

#### Dual-luciferase reporter assay and Chromatin immunoprecipitation (ChIP) assay

The dual-luciferase reporter assay and ChIP assay were carried out as previously described [20]. Briefly, HEK293T cells were plated on a 96-well plate and co-transfected with the *RGCC* promoter vectors and pcDNA3.1 ( +)-CEBPA or control vector. The pGL3-*RGCC* WT reporter or pGL3-*RGCC* MUT reporter was transfected into MDA-MB-231/LM3 cells with Lipofectamine 3000, and the Renilla luciferase reporter vector (PRL-TK vector) was used as a control. Following the transfection for 30 h, cell lysates were collected, and the luciferase activities of Renilla and Firefly were measured using a dual luciferase reporter assay system (Promega, USA). ChIP assays were carried out with the Pierce Agarose ChIP Kit (Cat. No. 26156, Thermo Fisher Scientific) using CEBPA and control IgG antibodies. The ChIP primer sequences are provided in Supplementary Table 3.

#### Methylation-specific PCR (MSP)

Cell genomic DNA was extracted following the manufacturer's instructions and modified with sulfite. Methylation-specific PCR amplification (MSP) was performed with 50 ng of sodium bisulfite-treated DNA, 200 ng of each primer, 1 × PCR buffer, 2.5 mM dNTPs, and 1 U of Taq polymerase (Dream Taq) in a 50 µl reaction system, and PCR products were subjected to agarose gel electrophoresis and photographed with a gel imager (Bio-Rad, USA). The methylation-specific primers and unmethylation-specific primers of CEBPA used in this study were designed through the online website of methprimer (http://www.urogene.org/methprimer/) and purchased from Sangon Biotech (Shanghai, China). All primers for the experiments are listed in Supplementary Table 4.

#### Western blotting analysis

Briefly, tumor cells and tumor tissues were lysed in RIPA buffer (Beyotime, China) to extract the total protein. Appropriate protein amount (50–80 μg/well) were subjected to SDS–PAGE and transferred to PVDF membranes (Bio-Rad, USA). The membranes were blocked with 5% defatted milk at room temperature for 1 h and incubated at 4 °C overnight with the specific primary antibodies against the following proteins: RGCC (1:1000; #30,125; SAB), CEBPA (1:500; ab40764; Abcam), PLK1 (1:1000; MG670393; Abmart), p-PLK1 (1:1000; #9062; Cell Signaling), AMPKα2 (1:500, 18,167–1-AP; proteintech), p-AMPK (1:1000; #50,081; Cell Signaling), ACC (1:1000; #3662; Cell Signaling), p-ACC (1:1000; #11,818; Cell Signaling), β-Actin (1:1000; Bioshap). The membranes were then incubated with horseradish peroxidase-coupled secondary antibodies for 2 h at room temperature, and images were captured using Scion image software. β-Actin was used as loading control. ImageJ software was used to quantify the intensity of the bands.

#### Immunohistochemistry (IHC)

IHC staining was performed using tissue sections from patients or mice according to the manufacturer's protocol. Briefly, tissue sections were dewaxed in xylene for antigen retrieval and blocked with goat serum, and then incubated with anti-RGCC (1:250) and anti-p-AMPK (1:250; #50,081; Cell Signaling) antibodies overnight at 4 °C. After incubating the sections with secondary antibodies at room temperature for 1 h, the sections were stained with a Diaminobenzidine (DAB) kit (Beyotime, China), and the images were captured by Nikon Eclipse 80i microscope (Tokyo, Japan).

#### Immunoprecipitation and LC–MS/MS

The immunoprecipitation assays were conducted using magnetic protein A/G immunoprecipitation beads (B23202, Bimake). The anti-RGCC and anti-PLK1 were used for immunoprecipitation. In short, the cells were centrifuged at 12,000 g for 30 min after being lysed on ice for 30 min with NP40 (P0013F, Beyotime). The supernatants were then collected, and a tiny quantity of lysate was used as the input group. The immunoprecipitation reaction was performed according to the manufacturer's protocol. Following immunoprecipitation, the beads were washed and the eluted proteins were analyzed by western blotting.

For LC–MS/MS analysis, MDA-MB-231/LM3 cells stably expressing RGCC were immunoprecipitated with anti-RGCC agarose beads. The products were enzymatically hydrolyzed overnight at 37 °C with an appropriate amount of 25 mM NH4HCO3 pH 8.0 enzymolysis buffer. After desalting the hydrolyzed peptide, the peptide samples were diluted to 1 μg/μl on the machine buffet, and the peptides in the sample were scanned. Mass spectral data were collected using the Triple TOF 5600 + LC/MS system (ABSCIEX, USA) and analyzed using a Triple TOF 5600 Plus mass spectrometer coupled to an Eksigent nanoLC system (AB SCIEX, USA). For protein identification, the Uniprot database was searched using the Paragon algorithm in Protein Pilot. Certain filter criteria were selected for the identified protein results, and the peptides with an unscore > 1.3 (over 95% confidence) were considered reliable peptides.

#### GST pull-down assay

GST pull-down assay was used to detect the direct interaction between RGCC and PLK1. Purified GST and GST-RGCC proteins were treated with recombinant HA-PLK1 for 4 h at 4 °C as part of the protein pull-down test. The mixture was then mixed with glutathione-Sepharose 4B beads and incubated for a further two hours at 4 °C. Nonidet P40 (NP-40) buffer (50 mM Tris–HCl, pH 7.4, 1% NP-40, 150 mM NaCl, 2 mM EDTA, 1 mM DTT, 10% glycerol) was used to wash the beads four times. Western blotting was carried out on the materials that had been bound with beads.

#### Metabolite profiling analysis

Metabolites were extracted from cell precipitation, and Waters ACQUITY UPLC BEH Amide (2.1 mm × 50 mm, 1.7 μm) was used for chromatographic separation of extracts. LC–MS/MS analyses were performed using an UHPLC system (Vanquish, Thermo Fisher Scientific). The raw data were converted into mzXML format using ProteoWizard software, and peak recognition, peak extraction, peak alignment, and integration processing were performed by a self-developed R program package, then the data were matched with the BiotreeDB (V2.1) self-built secondary mass spectrometry database for substance annotation. Six replicates per group were analyzed.

#### Transwell assay and cell viability assay

Cell invasion was measured as previously described [20]. In brief, tumor cells (2 × 10^4^) were seeded into the wells of 8-µm-pore Boyden chambers (Becton, Dickinson, USA) coated with Matrigel (1:7.5, Millipore, USA). After incubation for the indicated time, the transwell membrane was fixed with 4% paraformaldehyde, stained with 0.5% crystal violet, and the invaded cells were counted under a microscope.

For cell viability assay, cells were seeded onto 96-well plates at a density of 1000 cells per well and cultured for a specified time. Cell viability was measured using Cell Counting Kit-8 (Beyotime, China) following the manufacturer's description.

#### In vitro PLK1 kinase assay

The specific PLK1 kinase activity, with or without RGCC, was measured using the universal kinase activity kit (R&D Systems) according to the manufacturer's instructions. In a buffer consisting of 250 mM HEPES, 50 mM NaCl, 10 mM MgCl2, and 100 mM CaCl2, purified AMPKα2 and 1 mM ATP were combined with purified PLK1. The kinase reaction was carried out at 37 °C for 30 min and was terminated using malachite green reagent, and the optical density was measured at 600 nm to determine the amount of phosphorylated AMPKα2.

#### Mitochondrial membrane potential and oxygen consumption rate (OCR) measurement

For measurement of the mitochondrial membrane potential, the tumor cells on the confocal culture dishes were washed with PBS three times and incubated in JC-1 working solution (Beyotime, China) in the dark at 37 °C for 20 min. The images were acquired with an Eclipse Ti (Nikon, Japan), and the red fluorescence intensity (Ex/Em = 525/590 nm) representing JC-1 aggregates was measured with a Cary Eclipse (Agilent, USA).

For the measurement of oxygen consumption rate, the treatment of cells was referred to previous studies [21]. Cell culture medium was changed into XF basic culture medium before detection of OCR. After addition of 2 μM oligomycin, 1.5 μM carbonyl cyanide 4-(trifluoromethoxy) phenylhydrazone (FCCP), 2 μM rotenone, and 2 μM antimycin A, the OCR was determined by a Seahorse XF24 Extracellular Flux Analyzer (Seahorse Bioscience, USA).

#### Detection of fatty acid oxidation, NADPH and ROS

Fatty acid oxidation was detected with commercially available kits (ab222944, Abcam). In short, the cells were inoculated in 96-hole black plates and treated as directed by the manufacturer, and fluorescence was detected using FLUOstar Omega (BMG LABTECH, Elsbury, UK).

The ROS levels of cancer cells were detected using the ROS Assay Kit (Beyotime) following the manufacturer’s descriptions. Cells were washed with PBS and incubated for 20 min in the dark in serum-free medium containing 10 µM chloromethyl-2',7'-dichlorodihydrofluorescein (DCFH-DA). After removing the cell culture medium, DCFH-DA (S0033S, Beyotime) was added and incubated at 37 °C for 20 min. The fluorescence intensity (Ex/Em = 488/525 nm) was measured with a fluorescence enzyme marker.

The NADPH levels were determined using the NAPDH kit (S0179, Beyotime). Briefly, 1 × 10^6^ cancer cells were seeded and cultured in six-well plates for a set amount of time. Aspirate the culture medium and fill each well with 200 μl of NADP + /NADPH extracting solution. After incubating in the dark at 37 °C for 10 min, the absorbance at 450 nm was measured using an enzyme marker (Tecan, Switzerland) to calculate the amount of NADP + and the ratio of NADP + /NADPH.

#### Bioinformatics analysis

The microarray data consulted in this study were collected from Gene Expression Omnibus Datasets under accession numbers GSE14020, GSE32489, GSE5327, GSE32981, GSE34153, GSE74685, GSE157684 and GSE68468. Gene sets were obtained from GSEA (https://www.broadinstitute.org/gsea/), and gene set enrichment analysis (GSEA) was performed using the GSEA software.

#### Statistical analysis

All the experiments were repeated three times, and the data were expressed as the mean and standard deviation. Statistical analysis was performed by GraphPad Prism (version 8.0). Differences between groups were analyzed using a paired t test, two-way unpaired Student’s t test, or chi-square test. One-way analysis of variance (ANOVA) was performed for multiple groups. The Kaplan–Meier analysis was performed for the survival analysis of mice or patients. Correlations between RGCC and CEBPA expressions were examined using Pearson’s correlation coefficient. *P* < 0.05 was considered statistically significant.

## Results

### RGCC is highly expressed in lung metastases of TNBC cells and associated with TNBC prognosis

**Fig. 1:**
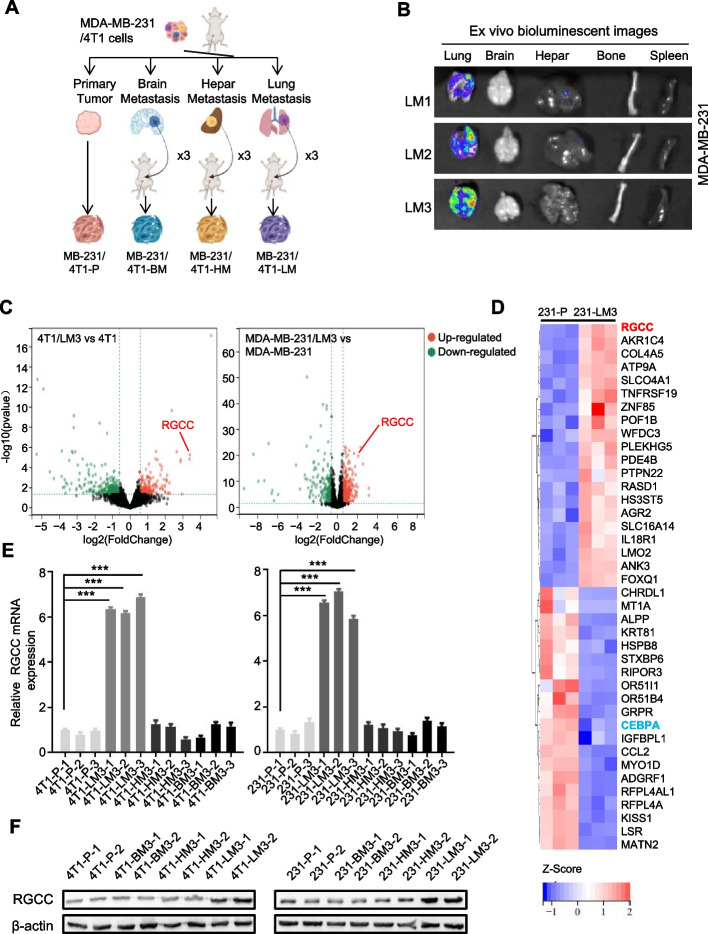
The enhanced RGCC in lung metastatic TNBC cells. **A** Schematic diagram to show the organotropic metastasis mouse model establishment of TNBC cells. **B** Representative bioluminescence images of lung-tropic metastasis of MDA-MB-231 cells in major organs. **C** Volcano plots of the altered gene expression between lung metastatic cells (LM3) and parental cells (P). The red and green dots represent the significantly upregulated and downregulated RNAs, respectively. **D** Heatmap shows 20 most upregulated or downregulated genes between MDA-MB-231/LM3 and MDA-MB-231/P cells. **E** Relative folds of RGCC mRNA expression in various metastatic organ sites were detected by qRT-PCR (P: parental; LM3/HM3/BM3: the third lung/liver/brain metastatic cells.). **F** Western blot analysis of RGCC protein levels in metastatic 4T1/MDA-MB-231 derived from various organ lesion and parental cells. (The data are presented as the mean ± SD; ^***^P < 0.001)

Extensive clinical studies have revealed that organ-specific metastases in solid tumors is the leading cause of cancer-related death. In order to establish experimental metastatic models of TNBC, the luciferase labeled TNBC cell lines 4T1 and MDA-MB-231 were used to generate organ-specific metastasis derivatives of TNBC through repeated fat pad injections and in vivo metastasis clone selection/expansion (Fig. 1A). The resulting subpopulations were named according to their source organs and generations. The LM3 derivatives (e.g. MDA-MB-231/LM3, the third-generation of lung metastatic cells) displayed lung-specific metastasis potential manifested by in vivo bioluminescence imaging (BLI) (Fig. 1B, Fig. S1A), suggesting the pulmonary-tropic metastasis mouse models of TNBC were established. To identify gene expression patterns associated with lung metastatic behavior, we performed RNA sequencing analysis of pulmonary-tropic metastatic cells (4T1-LM3, MDA-MB-231/LM3) vs their corresponding parental cells (4T1-P, MDA-MB-231-P)). As shown in the volcano plots (Fig. 1C), with cutoff thresholds of fold change > 2.0 and *P* < 0.05, a total of 181 genes were upregulated and 353 were downregulated in 4T1/LM3, while 381 genes were upregulated and 269 were downregulated in MDA-MB-231/LM3. 40 top changed genes were shown by heatmap; *RGCC* was most significantly changed in the metastatic derivatives compared with their parental cells (Fig. 1D, Fig. S1B). Notably, the expression of *RGCC* was solely detected in lung metastatic derivatives, but not in brain or hepatic metastases (Fig. 1E-F). In addition, the RGCC protein in lung metastatic cells was increased with the number of injections corresponding to the generated metastatic derivatives (Fig. S1C). Collectively, these data suggest a potential role of RGCC in promoting pulmonary-tropic metastasis of TNBC.

**Fig. 2:**
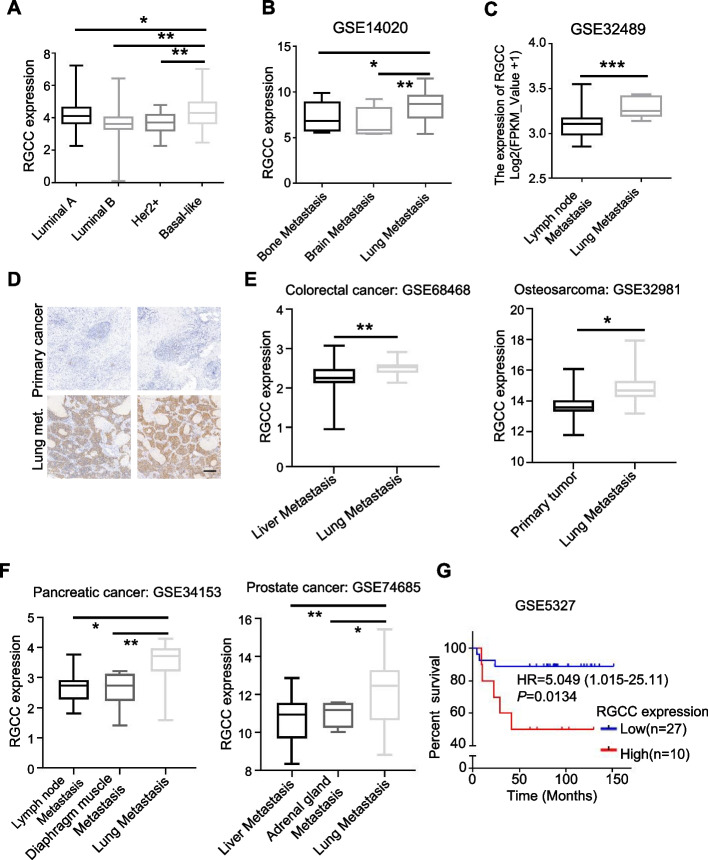
The increased RGCC is related with TNBC clinical features. **A-C** Analysis of RGCC expression in human breast cancer subtypes (A) and BC patients with different organ metastases (B, C) based on datasets from the TCGA database. In the boxplot, the middle line represents the median, and the bottom and top line correspond to the 25th and 75th percentiles. **D** Representative IHC images of RGCC in clinical tumor tissues of TNBC in site and lung metastases (Scale bars, 200 μm). **E**, **F** Increased RGCC mRNA expression was in lung metastases compared with other organs metastases or primary tumor based on the analysis of microarray datasets from different metastatic solid tumors. **G** Kaplan–Meier analysis to show patients’ survival of TNBC with high or low level of RGCC. (^*^P < 0.05, ^**^P < 0.01, ^***^P < 0.001)

To understand the clinical significance of RGCC in lung metastasis of breast cancer, we analyzed the expression of *RGCC* using TCGA database (RNAseq data) of breast cancer and found that *RGCC* had a higher expression level in malignant basal-like breast cancer compared with other breast cancer subtypes (Fig. 2A). Further analysis of the breast cancer cohort data in the Gene Expression Omnibus (GEO) revealed that the high expression level of *RGCC* was closely related with lung metastasis, but not metastasis to other organs (Fig. 2B-C). Similarly, the RGCC protein was overexpressed in breast cancer lung metastases of compared with primary tumor tissues (Fig. 2D). To extend our findings, we investigated *RGCC* expression in other tumors associated with lung-tropic metastasis from multiple public datasets. Interestingly, the increased *RGCC* expression was also found in lung metastases of multiple solid tumors, such as colorectal cancer, osteosarcoma, pancreatic cancer and prostate cancer (Fig. 2E-F). In addition, clinical statistical analysis revealed that the enhanced RGCC had a significant positive correlation with certain clinical features of TNBC patients, such as T stage (*P* = 0.0151), M stage (*P* = 0.0070) and TNM stage (*P* = 0.0257) (Table 1), and an inverse correlation with the overall survival in TNBC patients (Fig. 2G). Taken together, these data show that the increased expression level of RGCC is associated with breast cancer lung metastasis and poor prognosis in TNBC.

**Table 1:** The relationship between RGCC expression and clinical features in TNBC patients

Characteristics		All cases	RGCC		Chi-square value	*P* value
			**Low**	**High**		
All cases		68	34	34		
Age	< 50	28	13	15	0.2429	0.6222
	≥ 50	40	21	19		
T stage	T1	32	21	11	5.903	0.0151^*^
	T2/T3/T4	36	13	23		
N stage	N0/N1	37	22	15	2.905	0.0883
	N2/N3	31	12	19		
M stage	M0	29	20	9	7.275	0.0070^*^
	M1	39	14	25		
TNM stage	I/II	27	18	9	4.976	0.0257^*^
	III/IV	41	16	25		

^*^P < 0.05

### RGCC drives pulmonary-tropic metastasis of TNBC

**Fig. 3:**
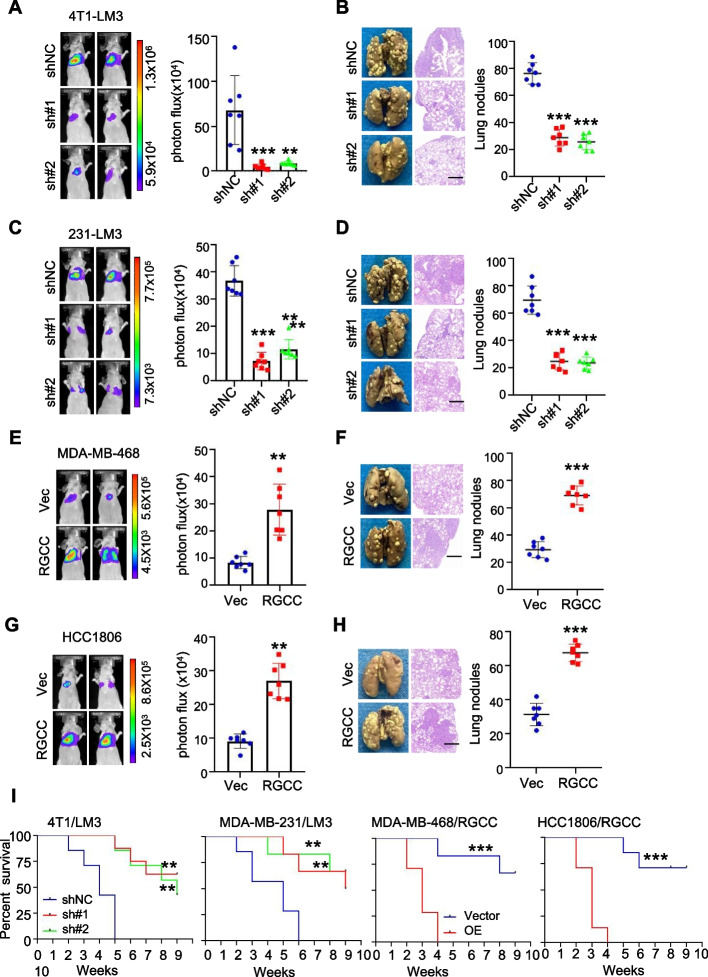
RGCC is a promoter for TNBC lung metastasis. **A-D** Intravenous injection of indicated TNBC cells with RGCC knockdown or control cells for 30 days, representative bioluminescence imaging (BLI) of lung metastasis (A, C) (*n* = 7 mice per group), pulmonary surface nodules, H&E images (B, D) (*n* = 7 mice per group) were shown (scale bars, 200 μm). **E–H** Experiments described as above, BLI (E) (*n* = 7 mice per group) (E, G), pulmonary surface nodules, H&E images (F, H) (*n* = 7 mice per group) in mice injected indicated TNBC cells with or without ectopic RGCC (scale bars, 400 μm). **I** Kaplan–Meier survival analyses of TNBC metastatic mice indicated as A-H (*n* = 7 mice per group). (The data are presented as the mean ± SD; ^**^P < 0.01, ^***^P < 0.001)

To access whether RGCC has a causal function in pulmonary-tropic metastasis, *RGCC* was stably knocked down in LM3 cells (4T1-LM3/RGCC KD, MDA-MB-231-LM3/RGCC KD) and overexpressed in selective TNBC cell lines (MDA-MB-468/RGCC, HCC1806/RGCC) (Fig. S2A-C) to examine its effect on metastasis in mice by bioluminescence imaging. Loss of RGCC in LM3 significantly reduced lung metastasis nodules (Fig. 3A-D) and prolonged lung metastases-bearing mice survival in comparison with control mice (Fig. 3I, left panels). Whereas ectopic expression of RGCC in MDA-MB-468 and HCC1806 led to more aggressive metastatic activity (Fig. 3E-H, and Fig. 3I, right panels). Consistent with the results that the increased RGCC promoted lung metastasis in vivo, RGCC-deficient cells had a notable decrease of cell invasion ability than the control cells (Fig. S2D-E) and ectopic RGCC overexpression increased cell invasion in vitro (Fig. S2F-G). Thus, these data demonstrate that RGCC is critical for lung metastasis of TNBC cancer cells.

### RGCC is negatively regulated by CEBPA

**Fig. 4:**
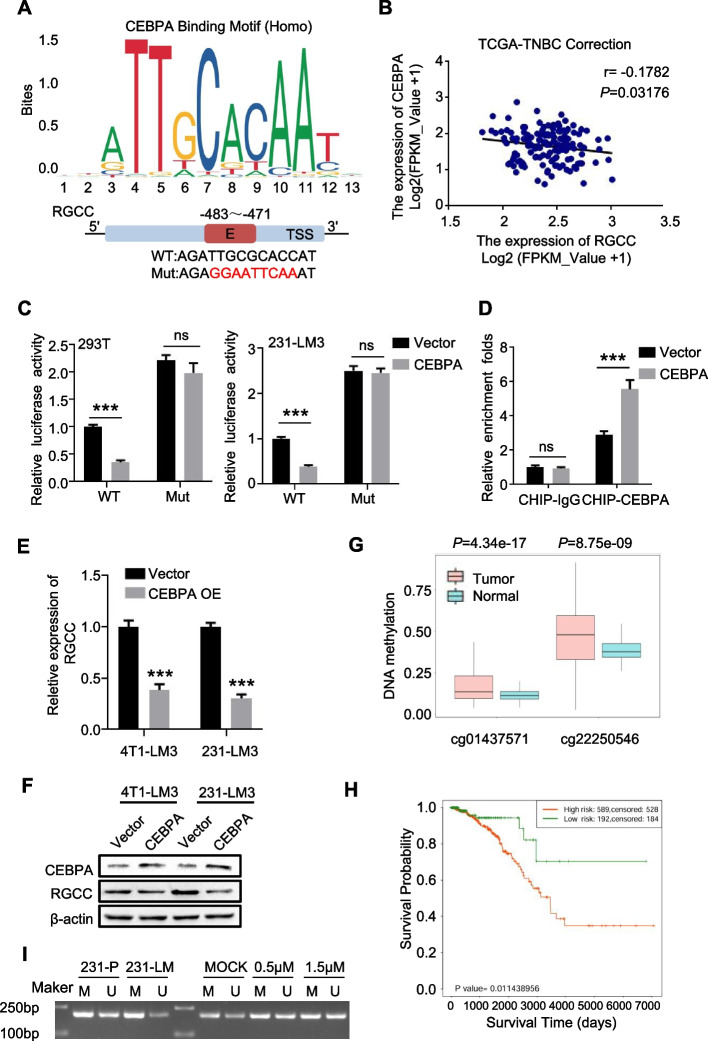
CEBPA negatively regulates RGCC expression. **A** The recognition motif of CEBPA in RGCC promoter (upper) analyzed using the JASPAR database, and the schematic illustration (down) showing the potential CEBPA responsive elements (E1). **B** A negative correlation between RGCC and CEBPA expression by Pearson correlation analysis using TCGA-TNBC dataset. **C** Luciferase reporter assays showing regulation of CEBPA regulation of RGCC promoter activity in 293 T and MDA-MB-231/LM3 cells. **D** ChIP assays to verify CEBPA binding affinity to the RGCC promoter in MDA-MB-231/LM3 transfected with CEBPA or control vector. **E**, **F** The regulation of CEBPA to RGCC was determined by qRT-PCR (E) and western blotting (F) in lung metastatic 4T1/LM3 and MDA-MB-231/LM3 cells. **G** High level of methylated CEBPA was in breast tumors than in normal tissues. **H** The Kaplan–Meier survival curves of BC patients with high or low methylated CEBPA. **I** Detection of methylation level in CpG islands of the CEBPA promoter by methylation specific polymerase chain reaction (MSP) (M: Methylation primer, U: Unmethylation primer.). (The data are presented as the mean ± SD; ^*^P < 0.05, ^**^P < 0.01, ^***^P < 0.001)

To explore the potential mechanism of upregulated RGCC in lung-specific metastases, we performed bioinformatics analysis to identify potential transcription factors (TFs) for the *RGCC* gene by the PROMO database (http://alggen.lsi.upc.es/) and RNA-seq data. The CEBPA was found to potentially regulate RGCC expression (Fig. S3A). CEBPA, a tumor suppressor [22, 23], was decreased in both LM3 derivatives (Fig. S3B). Further analysis using the JASPAR database (http://jaspar.genereg.net/) unveiled a potential CEBPA binding site (E1) in the *RGCC* promoter (Fig. 4A). Indeed, the *RGCC* level was negatively correlated with *CEBPA* in TCGA database (Fig. 4B). Using the luciferase reporter assay, we found that the *RGCC* promoter activity was obviously decreased in CEBPA overexpressing cells (Fig. 4C), which was further verified by chromatin immunoprecipitation analysis (ChIP) (Fig. 4D). Correspondingly, CEBPA overexpression significantly decreased *RGCC* mRNA (Fig. 4E) and protein expression (Fig. 4F) in 4T1-LM3 and MDA-MB-231/LM3 cells.

As a tumor suppressor, CEBPA is downregulated in multiple cancers [24], due to epigenetic regulation of DNA methylation [25]. Analysis of SurvivalMeth database, we found that there are two methylation sites at *CEBPA* promoter. Indeed, using the probes against these methylation sites, we confirmed that the methylation levels of *CEBPA* were markedly higher in breast tumors than in normal tissues (Fig. 4G). Furthermore, the breast cancer patients’ survival probability in high methylated *CEBPA* group was dramatically lower than that in low methylated *CEBPA* group (Fig. 4H), based on the best critical prognostic index analysis of a database from SurvivalMeth (Fig. S3C). Using methylation specific polymerase chain reaction detection (MSP), the hyper-methylation of *CEBPA* promoter was detected in MDA-MB-231/LM3 cells compared with their parental cells. Treatment of cells with Decitabine, a demethylated drug, could reduce the hyper-methylation level in MDA-MB-231/LM3 cells (Fig. 4I). Taken together, the decreased CEBPA in lung-specific metastases contributes to upregulation of RGCC.

### RGCC enhances PLK1 kinase activity

**Fig. 5:**
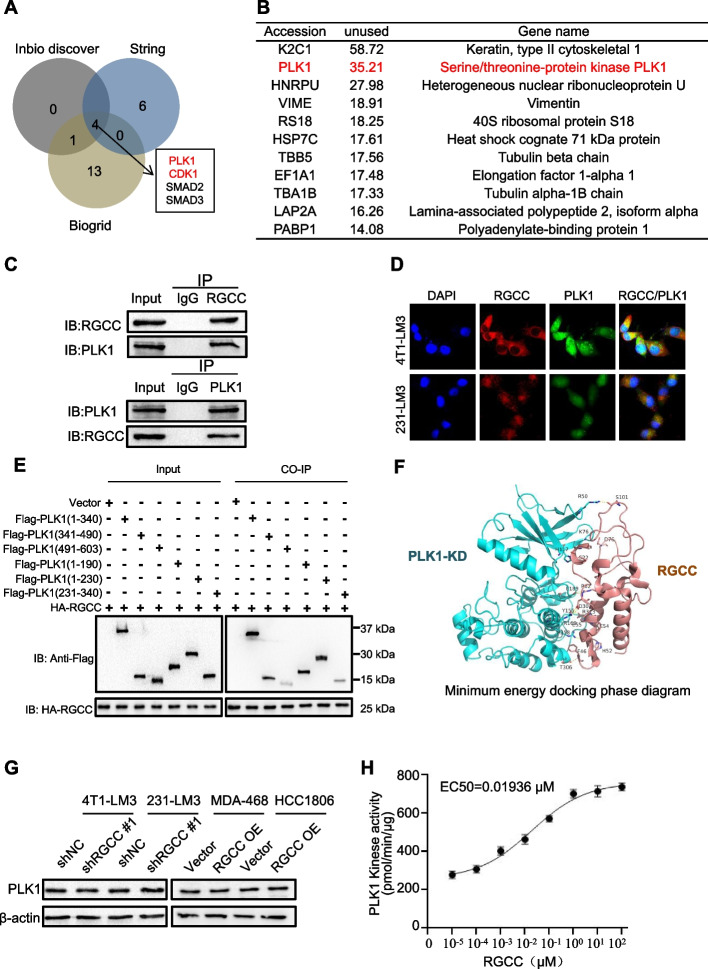
RGCC stimulates PLK1 kinase activity. **A** Venn diagram showing the potential interacting proteins of RGCC predicted by the database of Inbio discover, String and Biogrid. **B** Identification of the potential proteins interacting with RGCC by Mass Spectrometry. **C** Co-IP assays to confirm the direct interaction between RGCC and PLK1 in MDA-MB-231/LM3 cells using antibodies anti-RGCC or anti-PLK1, respectively. **D** IF co-staining showing the co-localization of RGCC (Red) and PLK1 (Green) in 4T1/LM3 and MDA-MB-231/LM3 cells. **E** Co-immunoprecipitation assay was used to detect the direct binding domain using HA-RGCC and FLAG-PLK1 truncation fragments in 293 T cells. **F** The binding mode of RGCC and PLK1 kinase domain (KD). **G** Western blot analysis of PLK1 protein levels under RGCC knockdown or overexpression. **H** PLK1 kinase assay showing RGCC affects PLK1 kinase activity. Data are means ± SD from 3 independent experiments

Previous studies have reported that RGCC binding with p34CDC2 protein kinase enhances the kinase activity [14]. To investigate the molecular mechanisms of RGCC function in lung-specific metastasis, bioinformatics analysis of Inbio discover, String, and Biogrid databases was carried out to predict target genes of RGCC. Four potential RGCC targets were found (Fig. 5A), including the protein kinases PLK1 and CDK1. Of note, CDK1 is a known target kinase of RGCC. Then, the RGCC-associated protein complex in MDA-MB-231/LM3 cells was isolated and identified through affinity purification followed by Liquid Chromatography-Tandem mass spectrometry. Among these, PLK1 was identified on the prey list (Fig. 5B). As expected, RGCC was bound with PLK1 in MDA-MB-231/LM3 cells (Fig. 5C) and 4T1/LM3 cells (Fig. S4A) by the co-immunoprecipitation assay. Immunofluorescent staining showed that RGCC and PLK1 were mainly co-localized in the cytoplasm of 4T1/LM3 and MDA-MB-231/LM3 cells (Fig. 5D). Furthermore, in vitro pulldown assays with purified recombinant proteins proved that RGCC can directly bind to PLK1 (Fig. S4B). The PLK1 protein is composed of a highly conserved N-terminal kinase catalytic domain (KD), two polo box domains (PBD) at the C-terminus, and an intermediate junction region [26]. To map the RGCC-interacting domain within PLK1, we constructed FLAG-tagged PLK1 deletion mutants, including PLK1 (1–340), PLK1 (341–490), PLK1 (491–603), PLK1 (1–190), PLK1 (1–230), and PLK1 (231–340). Using Co-IP analysis, RGCC was found to have strong binding to PLK1 (1–190) and PLK1 (341–490) fragments, and weak interaction with PLK1 (231–340) and PLK1 (491–603) fragments (Fig. 5E).

Previous studies have shown that the kinase activity of PLK1 is enhanced or weakened after direct interaction with certain proteins [27]. To understand whether RGCC can affect the kinase activity of PLK1, the binding of RGCC to PLK1 was firstly evaluated by the molecular docking and molecular dynamics simulation. The structure of protein complexes with the most favorable binding-free energies and reasonable orientations was selected as the optimal docked conformation. To further determine the stability of RGCC/PLK1-KD and RGCC/PLK1-PBD complexes, a 50 ns molecular dynamics simulation was performed. The result showed that the low root-mean-square deviation (RMSD) fluctuations and convergence of energy, temperature, and pressure were in the dynamics simulation system, indicating that RGCC/PLK1-KD and RGCC/PLK1-PBD complexes were stable systems at optimal docked conformation (Fig. S4C-D). Analysis of molecular docking found that RGCC protein could form a stable hydrogen bond interaction network with the non-substrate binding sites of the PLK1 active catalytic domain. The hydrogen bond interaction amino acid sites included HIS117 and ALA23, THR306 and PHE46, ARG158 and HIS52, and other amino acid residues (Fig. 5F). Additionally, RGCC protein could interact with PLK1 PBD, and the hydrogen bond interaction amino acid sites included LEU-491 and HIS-56, TYR-417 and ASP-36, PHE-535 and HIS-47, and other amino acid residues (Fig. S4E), which contribute to form the stable complexes between RGCC and PLK1. Of note, the expression of PLK1 had no significant change under knockdown or overexpression of RGCC (Fig. 5G). Phosphorylation of T210 is critical for PLK1 kinase activity both in vitro and in vivo. Western blotting was performed to test PLK1 and p-PLK1 (T210) levels in LM3 cells. Loss of RGCC led to a drastic reduction of p-PLK1, and RGCC overexpression induced higher p-PLK1 (Fig. S4F). Higher p-PLK1 protein levels were detected in lung metastatic derivatives compared with brain or hepatic metastases (Fig. S4G). These data indicate that the expression of RGCC is significantly correlated with the activity of PLK1, and supports the model in which RGCC stabilizes the active conformation of PLK1 by directly interacting with KD and PBD to release the T-loop, thus promoting the phosphorylation of PLK1 and subsequently activating PLK1 kinase (Fig. S4H). Under this premise, kinase activity was tested and the data showed that RGCC had a great effect on the kinase activity of PLK1, with a lower EC50 (the concentration required to reach the maximum activation of 50%) value (0.01936 μM) to PLK1 (Fig. 5H). Taken together, these data supported that RGCC can effectively promote the kinase activity of PLK1.

### RGCC/PLK1-mediated lung metastasis is dependent upon AMPKα2 activation

**Fig. 6:**
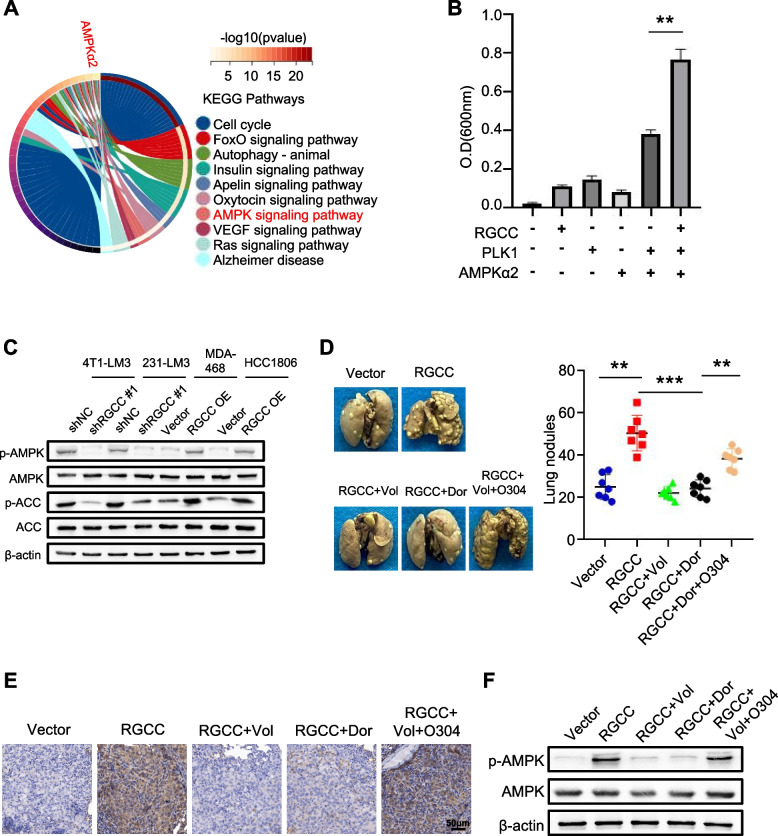
The RGCC/PLK1 axis activates AMPKα2. **A** The significant pathways associated with PLK1 were enriched by SangerBox based on the PLK1 potential phosphorylated proteins. **B** Determination of AMPKα2 phosphorylation in vitro. The experiments were independently repeated three times, with the error bars denoting the S.D. **C** Western blots of p-AMPK and p-ACC proteins in RGCC knockdown or overexpression tumor cells. **D** Orthotopic metastasis of RGCC overexpressed MDA-MB-468 cells treated with or without PLK1 inhibitor (Volasertb) or AMPK inhibitor (Dorsomorphin). Volasertib at 25 mg/kg, dorsomorphin at 0.2 mg/kg, or O304 at 200 mg/kg were given twice per week by oral gavage after the mouse’s tumor volume was above 50 mm^3^. Representative pulmonary surface nodules (n = 7 mice per group) are shown. **E** Immunostaining of p-AMPKα2 (T172) in lung metastatic tissues of mice with different treatments. **F** p-AMPKα2 (T172) protein levels in lung metastatic tissues of mice with different treatments were determined by western blotting

We further sought to investigate the downstream targets of the RGCC/PLK1 axis. The AMPK signaling pathway was a potential phosphorylated substrate of PLK1 after analysis of the phosphosite database (Fig. 6A). Previous studies also showed that PLK1 can phosphorylate AMPKα2 (the catalytic subunit of AMPK) [28], and phosphorylated AMPKα2 stimulates a strong AMPK signaling, which leads to metabolic reprogramming [29]. To test whether RGCC could promote the phosphorylation of AMPKα2 via PLK1, an in vitro kinase assay was carried out. The data revealed that the collaboration of RGCC and PLK1 significantly increased AMPKα2 phosphorylation (Fig. 6B). Of note, knockdown of RGCC led to a drastic reduction of AMPKα2 phosphorylation level. Transfection of ectopic PLK1 T210D (a hyper-phosphorylated PLK1 mutant), rather than PLK1 T210A (a hypo-phosphorylated PLK1 mutant), could restore AMPKα2 phosphorylation level in RGCC-deficient and endogenous PLK1 knockout tumor cells. In addition, by transfecting of ectopic PLK1 WT, PLK1 T210D into RGCC wild type and endogenous PLK1 knockout tumor cells, activation of AMPKα2 (p-AMPKα2) could be detected; however, transfection of ectopic PLK1 T210A into RGCC wild type and endogenous PLK1 knockout cells had a markedly reduced AMPKα2 phosphorylation level (Fig. S4I). Meanwhile, activation of PLK1 and AMPKα2 in RGCC-overexpression cells was impaired by Volasertib, a PLK1 kinase inhibitor (Fig. S4J). These data highlight the importance of RGCC-PLK1 axis in AMPK activation. Furthermore, we found that knockdown of RGCC led to a drastic reduction of p-AMPK and p-ACC, and RGCC overexpression induced higher levels of p-AMPK and p-ACC in TNBC cells (Fig. 6C).

To verify that the RGCC/PLK1 axis promotes lung metastasis by activating AMPKα2, RGCC-overexpressing MDA-MB-468 cells were used to test their metastasis potential *in vivo* by tail vein injection. Mice implanted with the RGCC-overexpressing MDA-MB-468 cells had increased lung metastases compared to those implanted with control MDA-MB-468 cells; however, MDA-MB-468/RGCC cell-injected mice under administration of Volasertib or Dorsomorphin (an AMPK activity inhibitor) had a significant reduction of lung metastases (Fig. 6D). Furthermore, mice injected with O304 (an AMPK activator) attenuated the Volasertib-caused reduction of the lung metastatic nodule number. Correspondingly, IHC staining and western blotting showed that the lung metastases from MDA-MB-468/RGCC tumor-bearing mice existed a significant activation of AMPK compared with the lung metastases from control mice; Volasertib or Dorsomorphin treatment of MDA-MB-468/RGCC tumor mice dramatically reduced the activity of AMPK in the lung metastasis, whereas simultaneous O304 and Volasertib treatment of MDA-MB-468/RGCC tumor mice partially restored the activity of AMPK in the lung metastasis (Fig. 6E-F). Collectively, these results reveal that enhanced RGCC expression leads to activation of AMPKα2 *in vivo* through RGCC-driven PLK1 activity to facilitate TNBC lung metastasis.

### RGCC/PLK1 activates AMPKα2 to coordinate mitochondrial oxidative phosphorylation and fatty acid oxidation for TNBC lung metastasis

**Fig. 7:**
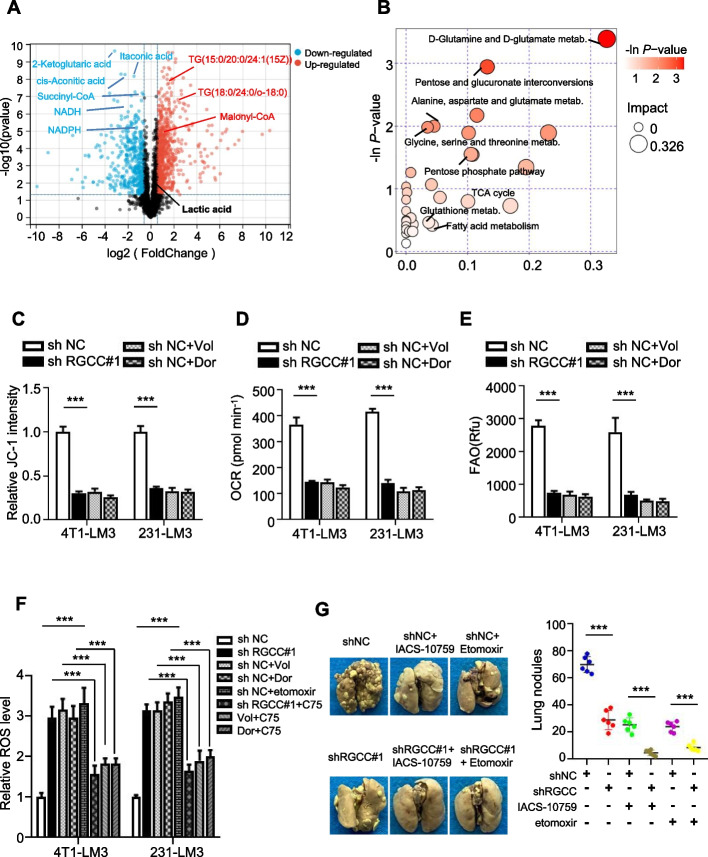
RGCC/PLK1/AMPKα2 signaling takes a role in TNBC lung metastasis. **A** Volcano plots of metabolites in RGCC knockdown MDA-MB-231/LM3 vs MDA-MB-231/LM3 control cells. **B** The enriched metabolic signaling pathways based on the genes regulating the high changed metabolites in control MDA-MB-231/LM3 cells. **C** Mitochondrial membrane potential was determined by JC-1 staining. **D** Oxygen consumption rate (OCR) was measured using a Seahorse XF24 Extracellular Flux Analyzer. **E** Fatty acid oxidation was detected using microplate reader. **F** ROS levels were measured by DCFH-DA probe. **G** Orthotopic metastasis of RGCC knockdown MDA-MB-231/LM3 cells was assessed in mice under treated with or without OXPHOS inhibitor (IACS-10759) or fatty acid oxidation inhibitor (Etomoxir). IACS-10759 at 25 mg/kg or Etomoxir at 20 mg/kg was given orally for 5 days followed by 2 days off every week, when the mice's tumor growth reached about 50 mm^3^. Representative pulmonary surface nodules (*n* = 7 mice per group) were shown. (Data are means ± SD from 3 independent experiments; ^***^P < 0.001)

As a classic energy sensor, AMPK involves in regulating metabolic reprogramming, allowing metastatic cancer cells to adapt to the harsh environment with metabolic stress and thrive at secondary sites [30]. This strongly suggests that RGCC/PLK1 may mediate lung metastasis of breast cancer through metabolic reprogramming via regulating AMPKα2. Thus, we identified metabolites in MDA-MB-231/LM3-sh*RGCC* and control cells through pan metabolomics GC/LC–MS analysis (Fig. S5A). A distinct metabolic profile was displayed in RGCC-knockdown MDA-MB-231/LM3 cells (Fig. S5B). The metabolomics study revealed markedly lower levels of itaconic acid, Succinyl-CoA, cis-aconitic acid, and α-Ketoglutarate in RGCC knockdown cells. These metabolites were intermediates of the tricarboxylic acid cycle (TCA cycle), which feed into OXPHOS (Fig. 7A). Additionally, there was no difference in lactate between MDA-MB-231/LM3-sh*RGCC* and MDA-MB-231/LM3-shNC cells (Fig. 7A). We further identified lower levels of NADPH (fatty acid oxidation products) and higher levels of lipids and malonyl-CoA (a highly regulatory molecule that can prevent fatty acid oxidation) in RGCC knockdown cells (Fig. 7A). Moreover, NADH, as the core of OXPHOS to generate energy in mitochondria, was decreased with the knockdown of RGCC (Fig. 7A). The pathway enrichment analysis showed that multiple amino acid metabolism was enriched in the metastatic cells, whose catabolism could also support TCA cycle and oxidative phosphorylation (Fig. 7B). We also observed enrichment of the metabolism of glutathione, fatty acid oxidation, and the pentose phosphate pathway (Fig. 7B), which were particularly pivotal for maintaining NADPH levels in cells to fuel survival. To analyze the RGCC associated gene expression profile using a GEO database (GSE157684), in which RGCC was knocked out in the cerebral organoid cohort, we found that RGCC may mainly involve in regulating AMPK and oxidative phosphorylation signaling pathways (Fig. S5C). Meanwhile, the enhanced RGCC was positively correlated with oxidative phosphorylation and fatty acid oxidation through GSEA analysis (Fig. S5D). Oxidative phosphorylation and fatty acid oxidation have been reported to promote the survival and metastasis of cancer cells [31]. Consequently, we raised a hypothesis that RGCC/PLK1/AMPKα2 might promote the lung colonization and metastasis of TNBC cells through regulating mitochondrial oxidative phosphorylation and fatty acid oxidation, respectively. In line with this, RGCC-deficient cells exhibited decreased mitochondrial activity (Fig. 7C), reduced mitochondrial oxygen consumption rate (OCR) (Fig. 7D), and lower basal respiration rate (Fig. S5E). Tumor cells treated with Volasertib or Dorsomorphin displayed a similar mitochondrial activity, OCR and basal respiration rate (Fig. 7D, Fig. S5E) as obtained in RGCC-deficient cells. RGCC knockdown tumor cells or tumor cells treated with Volasertib or Dorsomorphin also had a lower fatty acid oxidation level (Fig. 7E). In addition, RGCC knockdown cells or tumor cells treated with Volasertib, Dorsomorphin, or etomoxir (a fatty acid oxidation inhibitor) had a reduced NADPH/NADP + ratio (Fig. S5F) and increased ROS levels (Fig. 7F). Treatment with C75, a CPT1 activator that can increase fatty acid oxidation, could partially rescue the NADPH/NADP + ratio (Fig. S5F) and decreased ROS levels induced by Volasertib or Dorsomorphin (Fig. 7F). Correspondingly, the genes associated with oxidative phosphorylation (COX5B, NDUFS6, NDUFA8, CYC1, ATP5MF, UQCRH) and fatty acid oxidation (PPARα, CPT1, CPT2, ACOX1, ACOX2) were significantly downregulated in MDA-MB-231-LM3/sh*RGCC* cells compared to MDA-MB-231-LM3/shNC control cells. RGCC overexpression increased these gene expression in MDA-MB-468 cells (Fig. S5G). These data suggest that RGCC/PLK1/AMPKα2 signaling plays a crucial effect on OXPHOS and fatty acid oxidation for lung metastatic TNBC cells.

To understand whether RGCC/PLK1/AMPKα2 axis-mediated metabolic reprogramming could affect lung metastatic cell survival, and play a vital role in metastasis, lung metastases formation was assessed. As expected, RGCC knockdown, inhibition of OXPHOS with IACS-10759 (an oxidative phosphorylation inhibitor) or inhibition of fatty acid oxidation using etomoxir (a fatty acid oxidation inhibitor) reduced cell survival (Fig. S5H). Correspondingly, the incidences of lung colonization and metastasis were significantly reduced after IACS-10759 or etomoxir treatment in mice implanted with MDA-MB-231/LM3 cells. Knockdown of RGCC combined with IACS-10759 or etomoxir further reduced the pulmonary metastases (Fig. 7G). Collectively, RGCC/PLK1/AMPKα2 signal axis-mediated oxidative phosphorylation and fatty acid oxidation is critical for cell survival of disseminated tumor cells in lung, thereby facilitating pulmonary-tropic metastasis.

### Targeting RGCC combined with paclitaxel/carboplatin improves therapeutic benefits for lung-specific metastasis

**Fig. 8:**
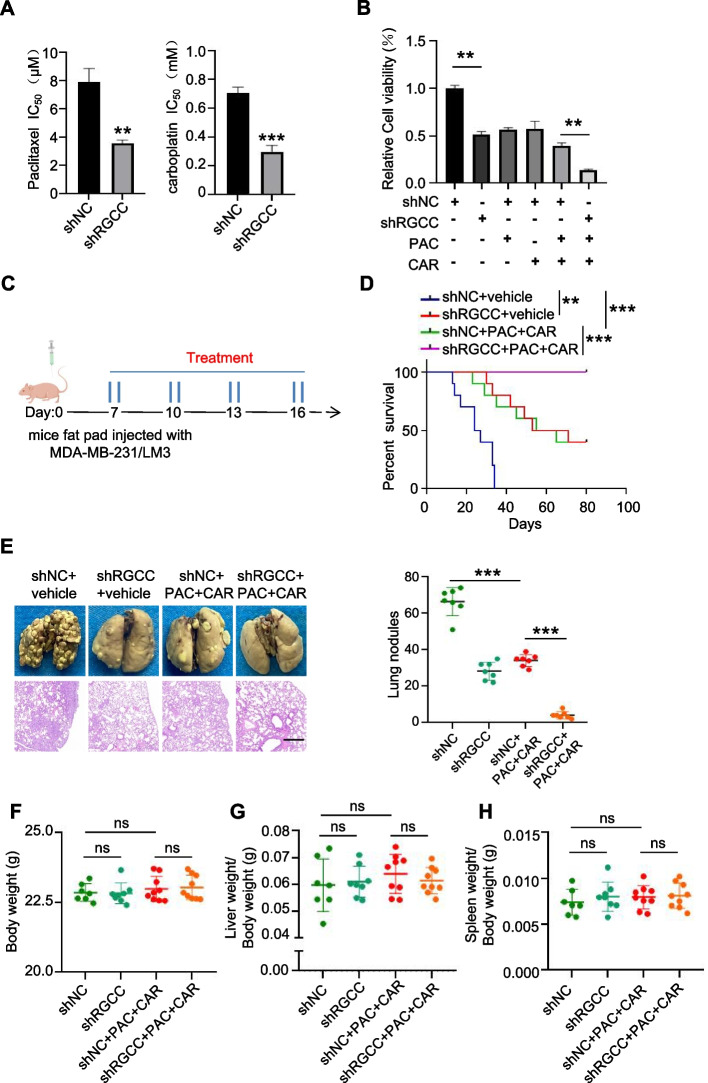
Targeting RGCC combined with paclitaxel/carboplatin therapy in lung-specific metastasis mice model. **A**,** B** Lung specific metastatic 4T1/LM3 and MDA-MB-231/LM3 cells with silencing RGCC and control cells were treated with serial dilutions of paclitaxel and carboplatin. IC50 values (**A**) and cell viability (**B**) were determined by CCK8 assay (*n* = 3). **C** Mice treatment scheme. **D** Overall survival of mice injected with MDA-MB-231/LM3/shNC or MDA-MB-231/LM3/shRGCC under combined management with or without paclitaxel/carboplatin (*n* = 7 mice per group). **E** Representative pulmonary surface nodules, H&E images (*n* = 7 mice per group) in each group of mice (scale bars, 400 μm). **F–H** Plots of the body weight (**F**), ratio of liver/body weight (**G**), and spleen/body weight (**H**) of mice (*n* = 7–9 mice/group). (Data were presented as mean ± SD; ^**^P < 0.01, ^**^.^*^P < 0.001, ns: no statistical difference)

Several studies have shown that carboplatin/paclitaxel combination therapy is the preferred treatment for metastatic breast cancer; however, the benefits of combined therapy are limited [32, 33]. We asked whether targeting RGCC could improve the therapeutic efficacies of the carboplatin paclitaxel combination regimen in treatment of TNBC lung metastasis. Indeed, administration of RGCC deficient MDA-MB-231/LM3 cells showed markedly decreased IC50 values of paclitaxel and carboplatin (Fig. 8A) and tumor cell viability (Fig. 8B) in contrast with RGCC wild type cells, suggesting that RGCC deficiency combined with paclitaxel and carboplatin treatment may have benefits for TNBC patients with lung metastasis. To further assess the efficiency of targeting RGCC in combination with carboplatin/paclitaxel, the mouse models injected with MDA-MB-231/LM3 RGCC-knockdown cells were used (Fig. 8C). Compared with control mice (labeled as MDA-MB-231/LM3/shNC/vehicle), carboplatin combined with paclitaxel treatment endowed mice with a prolonged survival and reduced pulmonary metastatic nodules. Knockdown of RGCC in combination with carboplatin/paclitaxel treatment further improved survival and reduced pulmonary metastatic nodules in these mice (Fig. 8D-E). No significant changes of body weight as well spleen and liver volumes of nude mice were observed, suggesting these treatments were well tolerated and had no significant toxicities (Fig. 8F-H). In conclusion, our findings show that the combination of carboplatin, paclitaxel, and RGCC inhibition can act synergistically to suppress breast cancer lung metastasis, which could bring therapeutic benefits for TNBC patients with lung metastasis.

## Discussion

The mortality of breast cancer patients is largely caused by metastasis [34]. Organotropism (organ specificity) is a substantial characteristic of tumor metastasis [35]. Despite a few groundbreaking studies carried out on organ-specific metastasis [36–38], the precise molecular mechanism of metastasis is not well understood. Here, we reveal that RGCC acts as a crucial player in lung metastasis of TNBC. RGCC is highly expressed in TNBC lung-specific metastatic cells and it enhances the activity of PLK1 kinase, leading to downstream AMPKα2 phosphorylation, thereby regulating oxidative phosphorylation and fatty acid oxidation. In a word, our work demonstrates that RGCC is a driving factor of lung-specific metastatic TNBC. The identified RGCC/PLK1/AMPKα2 axis is a potentially therapeutic target for TNBC lung metastasis.

The unique expression pattern of key genes or molecules in cancer cells and their selective interaction with the metastasis-permissive microenvironment may play a key role in distant organotropic metastasis of tumors. It was reported that LAPTM5 recruited WWP2 to mediate lysosomal degradation to drive lung metastases of renal cell carcinoma [39]. YTHDF3 is another organ-specific metastasis driver that regulates the translation of key metastasis genes such as T6GALNAC5, GJA1, EGFR, and VEGFA to promote breast cancer brain metastasis [37]. In this work, we uncover that RGCC is a crucial regulator and a diagnostic factor for TNBC lung-specific metastasis. In a comparison analysis of RGCC expression in TNBC patients with lung metastasis, the RGCC level in TNBC lung metastases tissue is higher than that in primary TNBC tissue. Although there are only a few paired specimens (primary tumor vs lung metastases), due to the difficulty of obtaining lung metastatic tissue of TNBC in clinical practice, it provides a valuable clue that RGCC is highly expressed in lung metastatic tissue of BC patients. In addition, the increased RGCC was found in lung metastases of colorectal cancer, osteosarcoma, pancreatic cancer, and prostate cancer in the GEO database. These imply that the upregulated RGCC is a common molecular event for multiple tumor types’ lung metastases, and may work as a diagnostic marker of pulmonary metastasis in patients with cancers. Thus, our work has implications for the screening of lung metastasis patients.

RGCC promotes breast cancer lung metastasis by regulating the kinase activity of PLK1. It is widely established that tumor development and metastasis are tightly tied to protein kinase's aberrant function. For instance, protein kinase D2 (PKD2) is implicated in the angiogenesis, metastasis, and invasion of various malignant tumors (e.g. liver cancer, lung cancer) after being activated [40]. It has been reported that tumor-derived factor (TDF) promotes the formation of a pre-metastatic lung microenvironment and the colonization of disseminated malignant cells in target organs by activating p38 kinase in lung fibroblasts [41]. Similarly, c-Src interacts with and phosphorylates the hexokinases HK1 and HK2, and phosphorylation of HK1 and HK2 significantly increases the kinase activity of hexokinase, thus promoting the occurrence and metastasis of tumors [42]. Hitherto, there have been few reports on the role of RGCC in tumor metastasis, especially how RGCC-induced kinase dysfunction affects metastasis. In this study, we confirmed that the highly expressed RGCC in lung metastatic derivative cells interacted with PLK1 to form a more stable complex and enhance its kinase activity to promote cancer metastasis, demonstrating that RGCC and PLK1 have important biological and diagnostic value in the process of lung metastasis.

The RGCC-PLK1-AMPKα2 axis was activated in TNBC lung metastases. AMPK, a heterotrimeric complex, can be activated under conditions of energy metabolism stress, allowing it to act as the energy sensor of cells [29]. Generally, the phosphorylation of threonine residue (Thr172) in AMPKα2 (AMPK complex subunit) increases the activity of AMPK [43]. Notably AMPK has been shown to act as a double edge sword for tumor development and metastasis, which may be influenced by distinct cellular contexts [44]. A recent study has found AMPK activation by metformin relieves the repressive H3K9me2-mediated silencing of epithelial genes during EMT processes, leading to inhibition of lung cancer metastasis [45]. Conversely, AMPK directly phosphorylates PDHA, which elicits PDHc activation and maintains TCA cycle to promote breast cancer metastasis [46]. Moreover, AMPK-mediated Skp2 S256 and AKT S473 phosphorylation promotes breast cancer progression in mouse tumor models and significantly correlates with metastasis-free survival in breast cancer patients [47]. Remarkably, we illuminated that activation of AMPKα2 serves as a downstream kinase of PLK1 necessary for oxidative phosphorylation and fatty acid oxidation in lung metastasis of TNBC.

Our findings reveal that RGCC-mediated oxidative phosphorylation is essential for lung metastasis. It’s well known that cancer cells rely on glycolysis rather than oxidative phosphorylation to generate energy and synthesize macromolecules even in the presence of oxygen [48]. However, a growing number of studies are starting to refute this dogmatic concept. Indeed, there is an increasing understanding that breast cancer [49], metastatic ovarian cancer [50], and glioblastoma [51] widely rely on mitochondrial oxidative phosphorylation as their primary energy source. In contrast to the higher concentration of glycolysis enzymes in primary tumor cells, mitochondrial oxidative phosphorylation (OXPHOS) may be a significant metabolic pattern during the disseminated tumor cell (DTC) seeding phase of lung metastasis. Additionally, the DTC seeding in lung tissues in mice was reduced by the pharmacological suppression of OXPHOS [49]. Indeed, previous studies have shown that the metabolic characteristics of breast cancer cells vary according to their metastatic sites. Bone and lung specific metastatic breast cancer cells rely on oxidative phosphorylation and mitochondrial biosynthesis, whereas the Warburg effect is observed in liver-specific metastatic breast cancer cells, which preferentially convert pyruvate to lactic acid [52]. Similarly, our findings suggest that OXPHOS is a common feature of lung-specific metastatic cancer cells. In a word, our findings provide new insight into the lung metastasis of TNBC cells.

Our research also unveils that the enhanced fatty acid oxidation is another metabolic adaptation pattern for TNBC lung metastasis. Fatty acid oxidation (FAO) is crucial for cells to overcome metabolic stress under conditions involving nutrient limitation by providing ATP and NADPH [53]. Cancer cells were found to facilitate fatty acid oxidation to increase NADPH levels and maintain various antioxidant defense systems by activating AMPK [54]. The link between FAO and tumor metastasis is supported by studies where the nuclear receptor Nur77-TPβ (a rate-limiting enzyme in FAO) interaction promotes melanoma metastasis by facilitating circulating melanoma cell survival [55]. Another study showed that FAO is also a critical metabolic adaptation for cancer metastasis to lymph nodes [56], as well as a drug target that regulates cellular state plasticity to drives breast cancer metastasis [57]. Consistent with recent findings that carnitine palmitoyl transferase 1A (CPT1A) mediated FAO activation increases the lung metastasis ability of colorectal cancer (CRC) cells [58], we confirmed that the RGCC-PLK1 mediated phosphorylation of AMPKα2 activates FAO, which is required for lung metastatic TNBC cells to maintain redox homeostasis and cell survival ultimately resulting in tumor metastasis. Furthermore, pharmacological suppression of oxidative phosphorylation and fatty acid oxidation decrease TNBC lung metastases.

The most common systemic therapy for metastatic breast cancer is still chemotherapy. However, the current treatment approaches are still not very effective in preventing recurrence or managing breast cancer metastasis because breast cancer exhibits metastasis heterogeneity and can metastasize to various organs, leading to differences in the prognosis and response to treatment of breast cancer patients [59]. Our study reveals that the overexpression of RGCC in TNBC cells that have spread to the lung, its activation of the PLK1-AMPK signal axis, regulation of OXPHOS and regulation of lipid acid oxidation, may offer a possible approach for clinically targeting TNBC lung metastasis. Importantly, targeting RGCC in combination with paclitaxel/carboplatin has conspicuous anti-metastasis effects, suggesting that reducing the expression of RGCC may be an efficacious treatment for lung metastases of TNBC.

## Conclusions

**Fig. 9:**
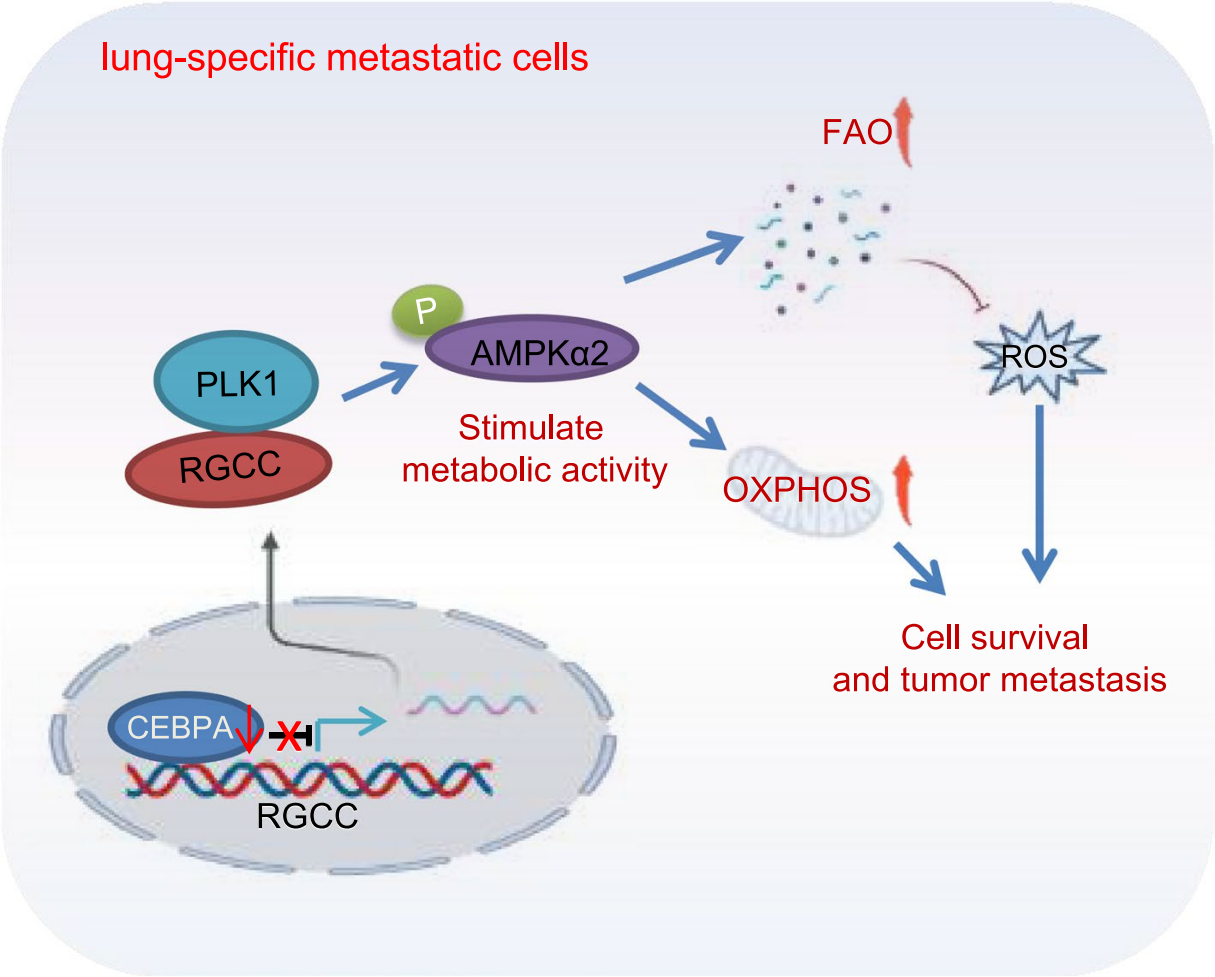
Schematic diagram displays the function of RGCC in promoting TNBC lung-tropic metastasis. The hyper-methylation of CEBPA (a transcription inhibitor) in lung-specific metastatic cancer cells leads to a reduced CEBPA and an increased RGCC. The enhanced RGCC interacts with PLK1 resulting in its higher kinase activity, phosphorylation of AMPKα2 and downstream mitochondrial oxidative phosphorylation and fatty acid oxidation activation, thus boosting the lung-tropic metastasis and colonization of TNBC

Herein, increased RGCC is closely associated with lung-specific metastasis of TNBC. RGCC leads to activation of AMPKα2 and downstream signaling through RGCC-driven PLK1 activity to facilitate TNBC lung metastasis, which may indicate a potential value of RGCC-driven OXPHOS and fatty acid oxidation as an important therapeutic target (Fig. 9).

## Availability of data and materials

The supporting data and materials are provided in Additional Figures and Tables.

### Supplementary Information

**Additional file 1:** **Supplemental Figure 1.** A. Representative bio-luminescence images of lung-tropic metastasis in mice injected with 4T1 cells into fat pad for 30 days. B. Heatmap showing 20 most upregulated and downregulated genes between 4T1/LM3 and 4T1 parental cells. C. Western blot analyses of RGCC expression in the indicated parental and derivative cells (231: MDA-MB-231). **Supplemental Figure 2.** A-C. Western blotting to assess RGCC knockdown efficiency in 4T1/LM3 and MDA-MB-231/LM3 cells (A), and RGCC proteins in the indicated TNBC cells (B), or in ectopic RGCC overexpressing MDA-MB-468 and HCC1806 cells (C). D-G. Transwell assay was used to evaluate the invasion ability of 4T1/LM3 and MDA-MB-231/LM3 cells transfected with shNC or shRGCC (D, E), and ectopic RGCC overexpressing MDA-MB-468 and HCC1806 cells (F, G) (Columns are the average of three independent experiments;^**^P < 0.01; 231: MDA-MB-231; MDA-468: MDA-MB-468). **Supplemental Figure 3.** A. A Venn diagram depicting the overlap transcriptional factors (TFs) between the altered TFs in MDA-MB-231/LM3 and the predicted TFs to potentially regulate RGCC expression by Promo Alggen database and JASPAR database. B. CEBPA mRNA levels in parental and derivative cells were determined by qRT-PCR. C. At the optimal cutoff prognostic index of 0.636, a total of 711 patient samples were divided into high risk (*n*=589) or low risk (*n*=192) group, according to the methylation level in CpG islands of CEBPA promoter. **Supplemental Figure 4.** A. Co-IP assays to confirm the direct interaction between RGCC and PLK1 in MDA-MB-231/LM3 cells using antibodies anti-RGCC or anti-PLK1, respectively. B. GST pulldown assay with purified HA-PLK1 and GST-RGCC to test the direct interaction between RGCC and PLK1. C-D. Molecular dynamics simulation assay to detect the stabilized binding between RGCC and PLK1 kinase domain (KD) (C), or between RGCC and PLK1 Polo-box domain (PBD) (D). The binding conformation of PLK1 was stabilized after a 22 ns simulation in (C) or a 20 ns simulation in (D). E. The binding mode of RGCC and PLK1 Polo-box domain (PBD). F-G. Western blot analysis of p-PLK1 (T210) protein levels in RGCC knocked down LM3 cells or RGCC overexpressing TNBC cells (F), and in various metastatic 4T1, MDA-MB-231 derived from brain, liver, lung and control parental cells (G). H. A proposed model explaining how RGCC promotes the intramolecular interaction between the PLK1 PBD and the kinase domain resulting in PLK1 kinase activity. I. WT or mutant (T210D, T210A) PLK1 was stably transfected into shNC or shRGCC with endogenous PLK1 knockout tumor cells, western blotting was used to determine p-AMPKα2 levels. J. Western blotting to check p-PLK1 and p-AMPKα2 levels in RGCC-overexpression cells by administration of Volasertib (a PLK1 kinase inhibitor). **Supplemental Figure 5.** A. Principal component analysis (PCA) of metabolites shows the percentage of variation for the first and second principal components. B. Heatmap analysis of significantly changed metabolites between RGCC-engineered MDA-MB-231/LM3 cells (sh RGCC vs sh NC) (*n*=6). C. The major signaling related with RGCC revealed by SangerBox analysis using microarray datasets GSE157684. D. Gene set enrichment analysis (GSEA) displays the relationship between RGCC expression and fatty acid oxidation and oxidative phosphorylation. E. Basal respiration of the indicated LM3 cells was detected using a Seahorse XF24 Extracellular Flux Analyzer. F. NADPH/NADP^+^ ratio of the indicated LM3 cells was detected using microplate reader. G. qRT-PCR was used to detect the levels of genes associated oxidative phosphorylation and fatty acid oxidation in RGCC knockdown MDA-MB-231/LM3 and RGCC overexpressing MDA-MB-468 cells. H. Cell viability was evaluated by CCK8 assay. (Data were presented as mean ± SD; ^**^P < 0.01,^***^P < 0.001).

**Additional file 2:** **Supplemental Table 1.** Core sequences of shRNA against target genes. **Supplemental Table 2.** List of primer sequences utilized in the study. **Supplemental Table 3.** Primer sequences used for PCR analysis in ChIP assay.**Supplemental Table 4.** Methylation and Unmethylation specific primers.

## Abbreviations

TNBC: Triple negative breast cancer
RGCC: Completion 32 protein response gene
OXPHOS: Oxidative phosphorylation
FAO: Fatty acid oxidation
OCR: Oxygen consumption rate
GSEA: Gene Set EnrichmentAnalysis
shRNA: Short-hairpin RNA
KEGG: Kyoto Encyclopedia of Genes and Genomes
ROS: Reactive oxygen species
