# Supplementary figures and images for: RGCC-mediated PLK1 activity drives breast cancer lung metastasis by phosphorylating AMPKα2 to activate oxidative phosphorylation and fatty acid oxidation
Source: J Exp Clin Cancer Res. 2023 Dec 15;42:342. doi: 10.1186/s13046-023-02928-2 (PMC10722681; doi:10.1186/s13046-023-02928-2)

A

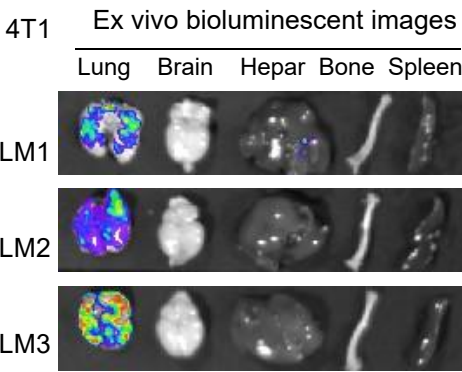

B

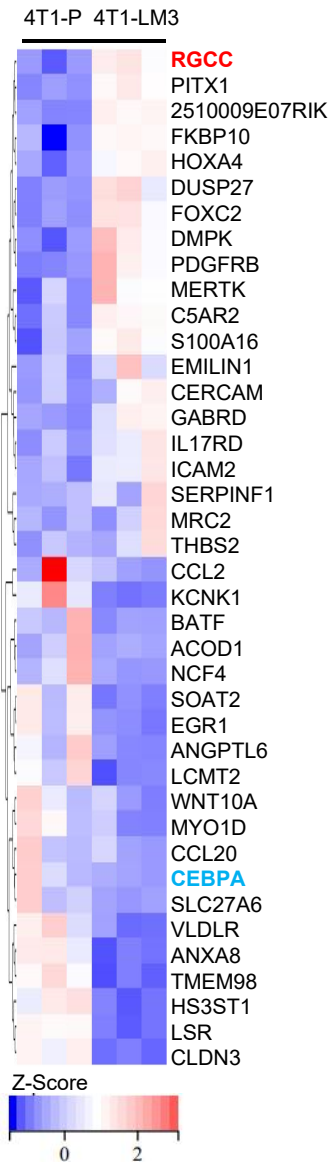

C

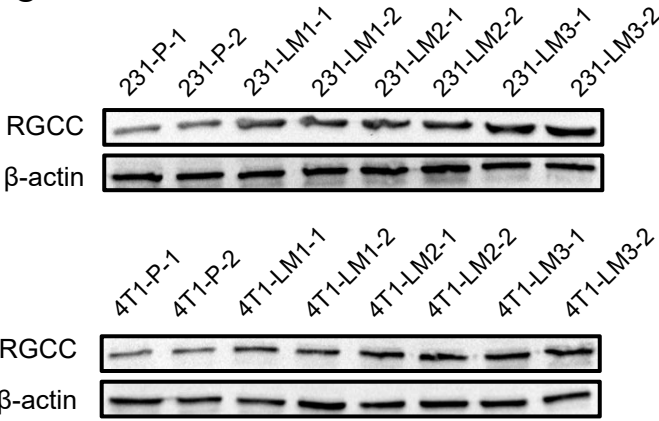

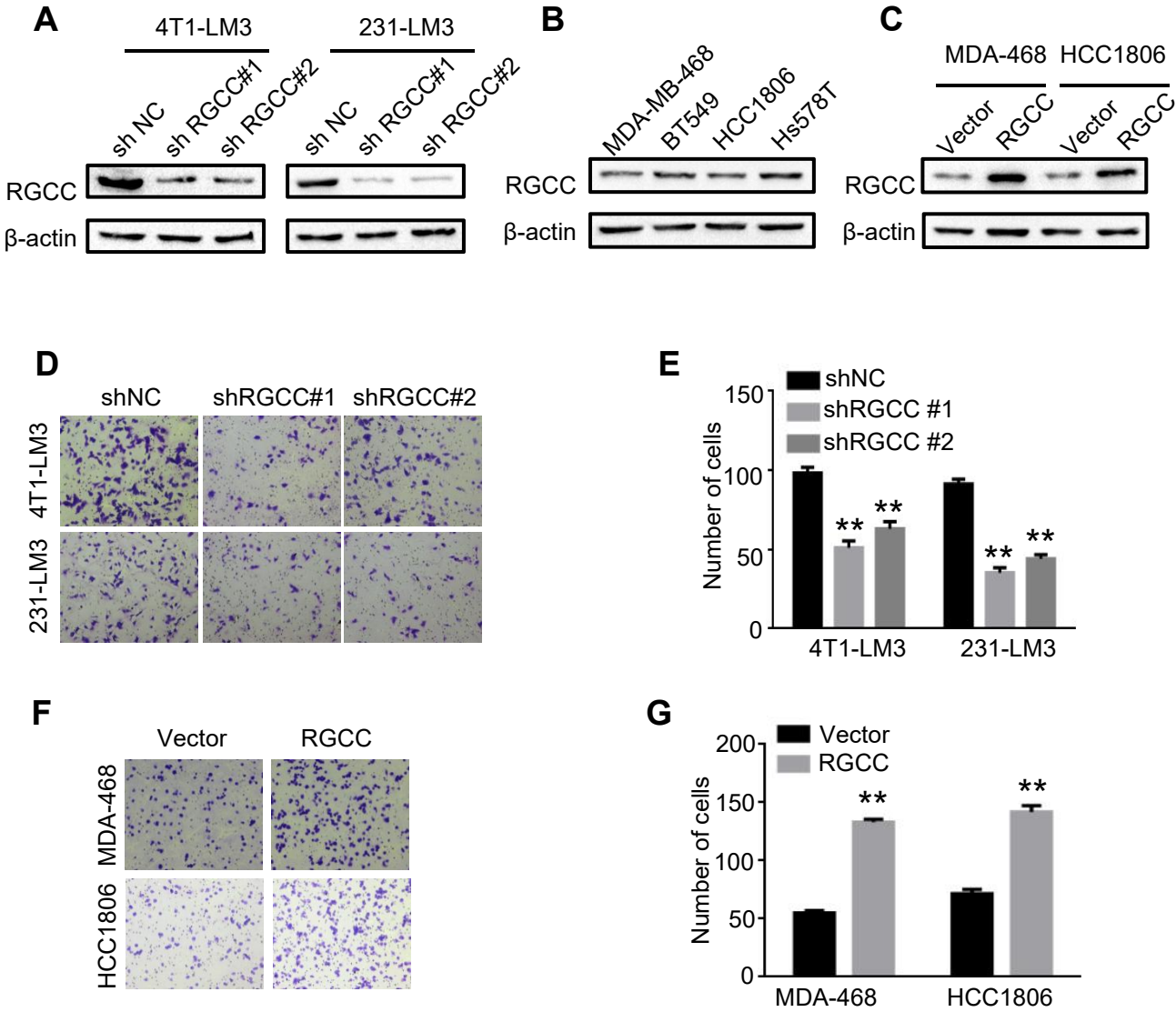

A

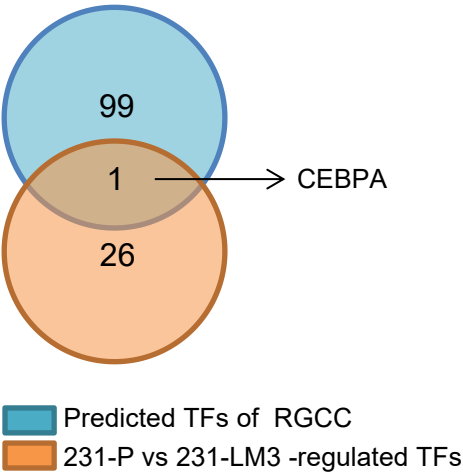

B

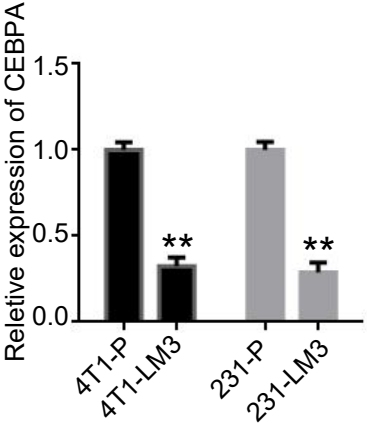

C

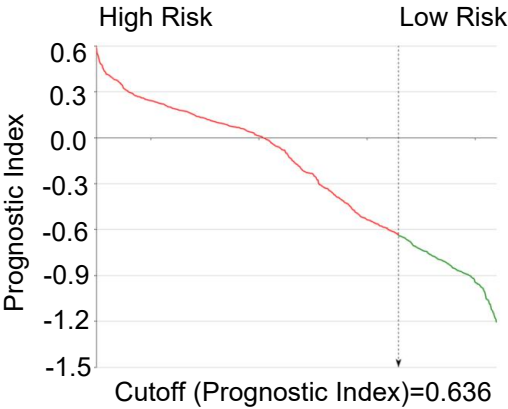

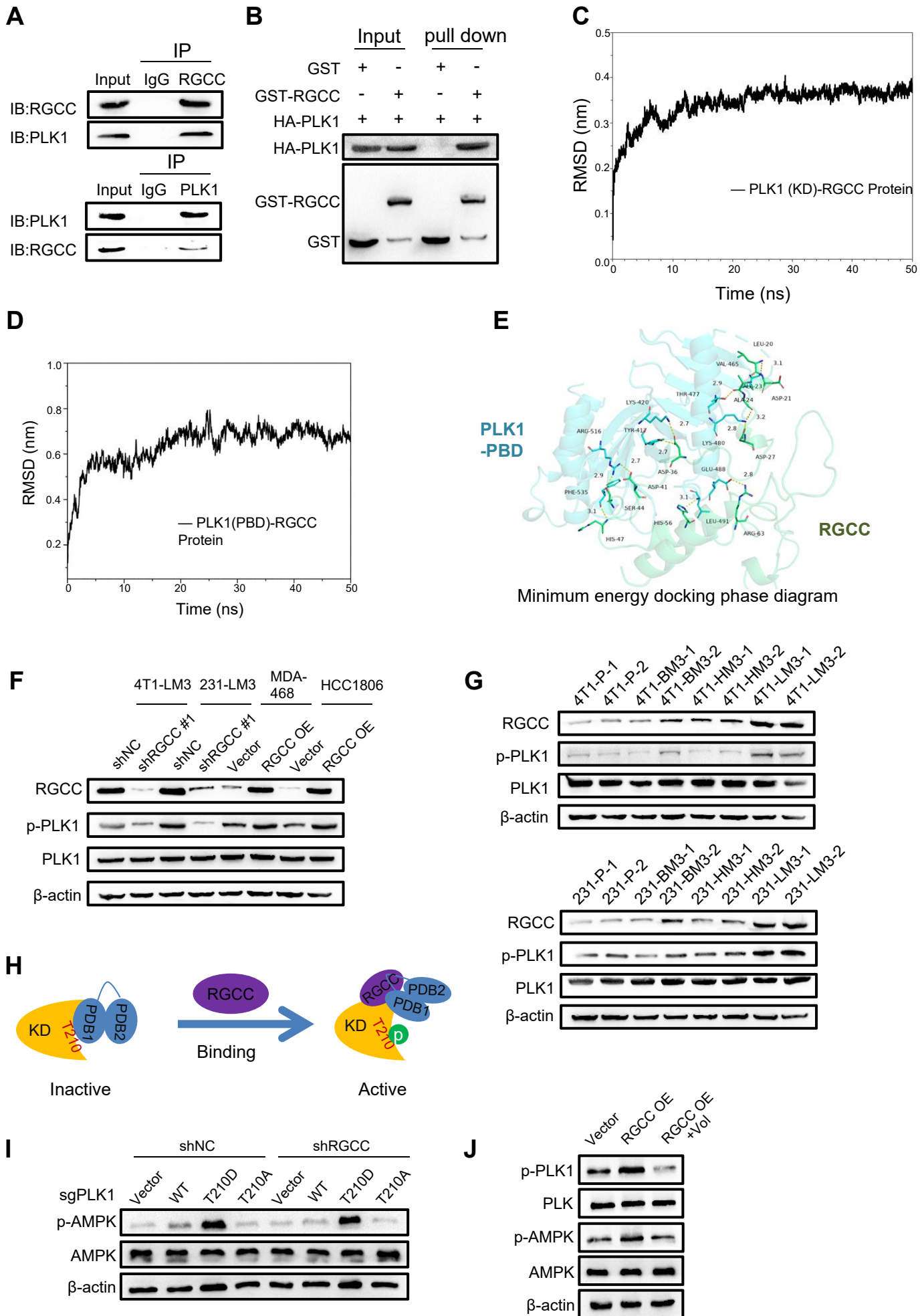

A

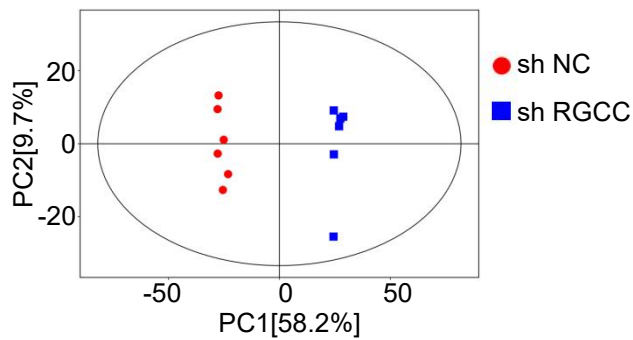

B

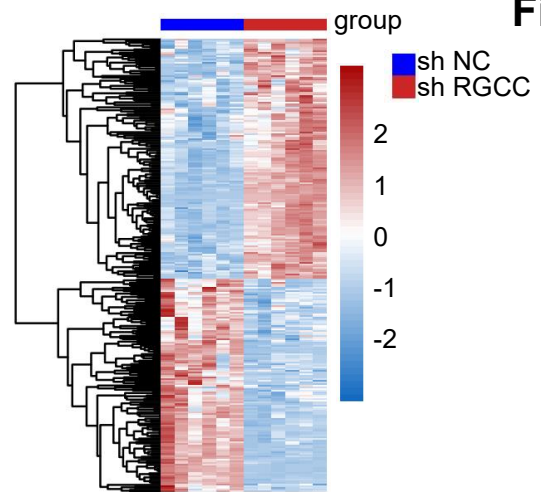

C

RGCC-KO\_WT.diff.down.Pathway.enrichment

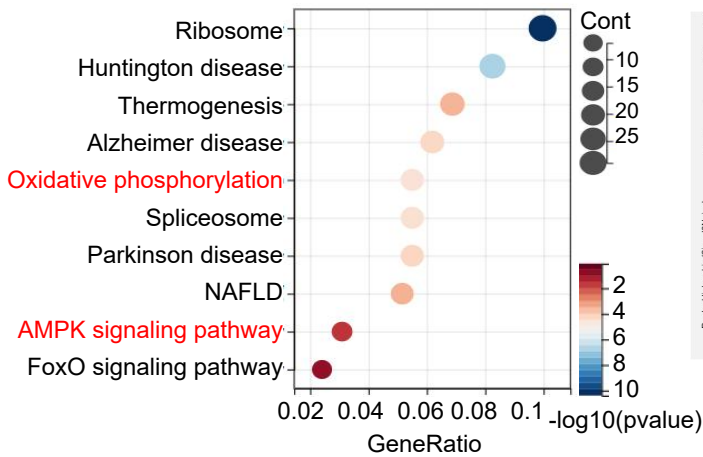

D

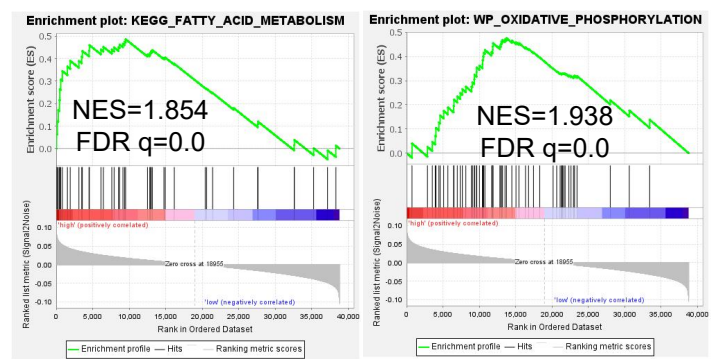

E

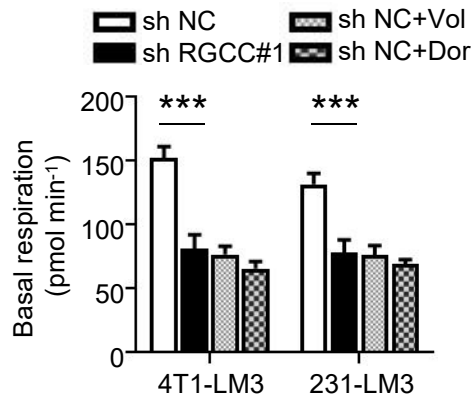

F

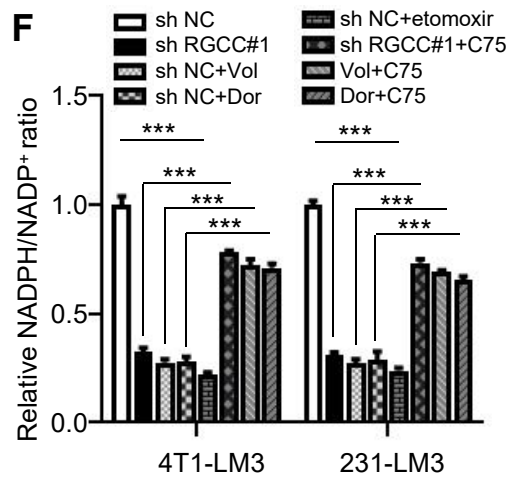

G

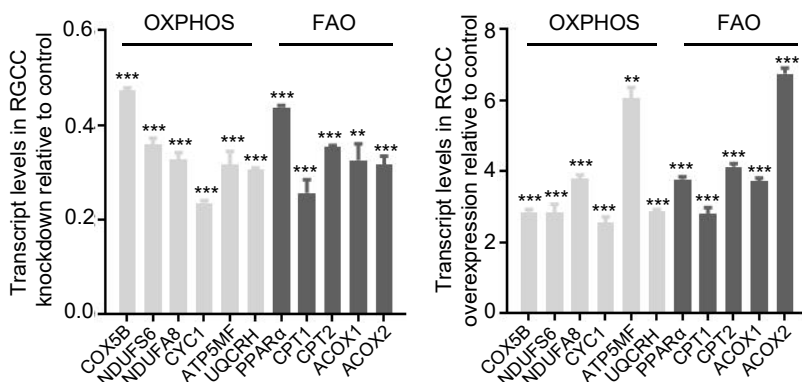

H

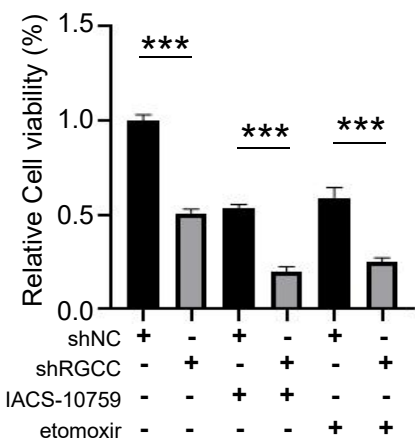

Supplement: Supplementary file 1 — Additional file 1: Supplemental Figure 1. A. Representative bio-luminescence images of lung-tropic metastasis in mice injected with 4T1 cells into fat pad for 30 days. B. Heatmap showing 20 most upregulated and downregulated genes between 4T1/LM3 and 4T1 parental cells. C. Western blot analyses of RGCC expression in the indicated parental and derivative cells (231: MDA-MB-231). Supplemental Figure 2. A-C. Western blotting to assess RGCC knockdown efficiency in 4T1/LM3 and MDA-MB-231/LM3 cells (A), and RGCC proteins in the indicated TNBC cells (B), or in ectopic RGCC overexpressing MDA-MB-468 and HCC1806 cells (C). D-G. Transwell assay was used to evaluate the invasion ability of 4T1/LM3 and MDA-MB-231/LM3 cells transfected with shNC or shRGCC (D, E), and ectopic RGCC overexpressing MDA-MB-468 and HCC1806 cells (F, G) (Columns are the average of three independent experiments;**P < 0.01; 231: MDA-MB-231; MDA-468: MDA-MB-468). Supplemental Figure 3. A. A Venn diagram depicting the overlap transcriptional factors (TFs) between the altered TFs in MDA-MB-231/LM3 and the predicted TFs to potentially regulate RGCC expression by Promo Alggen database and JASPAR database. B. CEBPA mRNA levels in parental and derivative cells were determined by qRT-PCR. C. At the optimal cutoff prognostic index of 0.636, a total of 711 patient samples were divided into high risk (n=589) or low risk (n=192) group, according to the methylation level in CpG islands of CEBPA promoter. Supplemental Figure 4. A. Co-IP assays to confirm the direct interaction between RGCC and PLK1 in MDA-MB-231/LM3 cells using antibodies anti-RGCC or anti-PLK1, respectively. B. GST pulldown assay with purified HA-PLK1 and GST-RGCC to test the direct interaction between RGCC and PLK1. C-D. Molecular dynamics simulation assay to detect the stabilized binding between RGCC and PLK1 kinase domain (KD) (C), or between RGCC and PLK1 Polo-box domain (PBD) (D). The binding conformation of PLK1 was stabilized after a 22 ns sim [file 13046_2023_2928_MOESM1_ESM.pdf]
