# Supplementary material for: RGCC-mediated PLK1 activity drives breast cancer lung metastasis by phosphorylating AMPKα2 to activate oxidative phosphorylation and fatty acid oxidation
Source: J Exp Clin Cancer Res. 2023 Dec 15;42:342. doi: 10.1186/s13046-023-02928-2 (PMC10722681; doi:10.1186/s13046-023-02928-2)
Supplement: Supplementary file 2 — Additional file 2: Supplemental Table 1. Core sequences of shRNA against target genes. Supplemental Table 2. List of primer sequences utilized in the study. Supplemental Table 3. Primer sequences used for PCR analysis in ChIP assay.Supplemental Table 4. Methylation and Unmethylation specific primers. [file 13046_2023_2928_MOESM2_ESM.docx]

| **Supplemental Table 1. Core sequences of shRNA against target genes** | | |
| --- | --- | --- |
| **Target gene** | **Target sequences** |  |
| Rgcc#1(Mus) | 5’-GCCAAATTAGGTGACACTAAA-3’ |  |
| Rgcc#2(Mus) | 5’-CGAAGACTTCATTGCCGATCT-3’ |  |
| RGCC#1(Homo) | 5’-CCUGCAGUUUGAUGCUGAUTT-3’ |  |
| RGCC#2(Homo) | 5’-CAGAUUCACUUUAUAGGAA-3’ |  |

**Supplemental Table 2.** **List of primer sequences utilized in the study**

| **Gene name** | **Primer sequences** |
| --- | --- |
| RGCC (Homo) | F: 5’-AACAGACTCTACCCCAGCTCT-3’  R: 5’-GGCTTCTAGCTCTTTTGTGTCT-3’ |
| Rgcc (Mus) | F: 5’-CTCCAACCAACTCCTCTCC-3’  R: 5’-GACCCCAAACTCCTTGCT-3’ |
| CEBPA (Homo) | F: 5’-GCCAAGAAGTCGGTGGACAA-3’  R: 5’-ATTGTCACTGGTCAGCTCCA-3’ |
| Cebpa (Mus) | F: 5’-GAGCCCCCTCTGAGTCAG-3’  R: 5’-GCAAAAACATCTTGTTGAG-3’ |
| COX5B (Homo) | F: 5’-ATGGCTTCAAGGTTACTTCGC-3’  R: 5’-CCCTTTGGGGCCAGTACATT-3’ |
| NDUFS6 (Homo) | F: 5’-TTCGGTTTGTAGGTCGTCAGA-3’  R: 5’-CCATCGCACGCTATCACCC-3’ |
| NDUFA8 (Homo) | F: 5’-CCCAACAAGGAGTTTATGCTCT-3’  R: 5’-CACAGTGACGTTTTATCTGCCT-3’ |
| CYC1 (Homo) | F: 5’-CTTCGCGGGGTAGTGTTGG-3’  R: 5’-GGCCAGACTTCGACGACAA-3’ |
| NDUFB10 (Homo) | F: 5’-AGCCCAATCCCATCGTCTACA-3’  R: 5’-GCTGCCGCTCTATAAATTCTCT-3’ |
| UQCRH (Homo) | F: 5’-GAGGACGAGCAAAAGATGCTT-3’  R: 5’-CGAGAGGAATCACGCTCATCA-3’ |
| PPARα (Homo) | F: 5’-TTCGCAATCCATCGGCGAG-3’  R: 5’-CCACAGGATAAGTCACCGAGG-3’ |
| CPT1 (Homo) | F: 5’-ATGCGCTACTCCCTGAAAGTG-3’  R: 5’-GTGGCACGACTCATCTTGC-3’ |
| CPT2 (Homo) | F: 5’-CTGGAGCCAGAAGTGTTCCAC-3’  R: 5’-AGGCACAAAGCGTATGAGTCT-3’ |
| ACOX1 (Homo) | F: 5’-ACTCGCAGCCAGCGTTATG-3’  R: 5’-AGGGTCAGCGATGCCAAAC-3’ |
| ACOX2 (Homo) | F: 5’-GCACCCCGACATAGAGAGC-3’ R: 5’-CTGCGGAGTGCAGTGTTCT-3’ |
| β-actin (Homo) | F: 5'-GCCGAGGACTTTGATTGC-3’  R: 5'-CCTGTGTGGACTTGGGAGA-3’ |
| β-actin (Mus) | F: 5’-GCTATGCTCTCCCTCACG-3’  R: 5’-ACGCACGATTTCCCTCT-3’ |

| **Supplemental Table 3. Primer sequences used for PCR analysis in ChIP assay** | |
| --- | --- |
| **Gene name** | **Primer sequences** |
| RGCC | F: 5’-AGATTGCGCACCATTAGAAGTGGGT-3’  R: 5’-TTAGAAACAAGGGACCTGTCCAGGC-3’ |

**Supplemental Table 4. Methylation and Unmethylation specific primers**

| **Gene name** | | **Primer sequences** | | | | |
| --- | --- | --- | --- | --- | --- | --- |
| CEBPA-Left-M | | | TTGGAGATTAGAGTTAGGAGACGT |  |  |  |
| CEBPA-right-M | | | ACACGAAATAAAAATAAAAAACGAA |  |  |  |
| CEBPA-Left-U | | | TTTGGAGATTAGAGTTAGGAGATGT |  |  |  |
| CEBPA-right-U | | | | CACAAAATAAAAATAAAAAACAAA | | |

**M:Methylation primer; U: Unmethylation primer**
